# Comparative Metagenomics and Network Analyses Provide Novel Insights Into the Scope and Distribution of β-Lactamase Homologs in the Environment

**DOI:** 10.3389/fmicb.2019.00146

**Published:** 2019-02-11

**Authors:** Joao Gatica, Edouard Jurkevitch, Eddie Cytryn

**Affiliations:** ^1^Institute of Soil, Water and Environmental Sciences, Volcani Center, Agricultural Research Organization, Rishon LeZion, Israel; ^2^The Department of Soil and Water Sciences, The Robert H. Smith Faculty of Agriculture, Food and Environment, The Hebrew University of Jerusalem, Rehovot, Israel; ^3^Department of Plant Pathology and Microbiology, The Robert H. Smith Faculty of Agriculture, Food and Environment, The Hebrew University of Jerusalem, Rehovot, Israel

**Keywords:** antibiotic resistance, β-lactamases, network analysis, metagenomics, environment

## Abstract

The β-lactams are the largest group of clinically applied antibiotics, and resistance to these is primarily associated with β-lactamases. There is increasing understanding that these enzymes are ubiquitous in natural environments and henceforth, elucidating the global diversity, distribution, and mobility of β-lactamase-encoding genes is crucial for holistically understanding resistance to these antibiotics. In this study, we screened 232 shotgun metagenomes from ten different environments against a custom-designed β-lactamase database, and subsequently analyzed β-lactamase homologs with a suite of bioinformatic platforms including cluster and network analyses. Three interrelated β-lactamase clusters encompassed all of the human and bovine feces metagenomes, while β-lactamases from soil, freshwater, glacier, marine, and wastewater grouped within a separate “environmental” cluster that displayed high levels of inter-network connectivity. Interestingly, almost no connectivity occurred between the “feces” and “environmental” clusters. We attributed this in part to the divergence in microbial community composition (dominance of *Bacteroidetes* and *Firmicutes* vs. *Proteobacteria*, respectively). The β-lactamase diversity in the “environmental” cluster was significantly higher than in human and bovine feces microbiomes. Several class A, B, C, and D β-lactamase homologs (*bla*_CTX-M_, *bla*_KPC_, *bla*_GES_) were ubiquitous in the “environmental” cluster, whereas bovine and human feces metagenomes were dominated by class A (primarily *cfxA*) β-lactamases. Collectively, this study highlights the ubiquitous presence and broad diversity of β-lactamase gene precursors in non-clinical environments. Furthermore, it suggests that horizontal transfer of β-lactamases to human-associated bacteria may be more plausible from animals than from terrestrial and aquatic microbes, seemingly due to phylogenetic similarities.

## Introduction

The β-lactam antibiotics are the most widely used clinically applied antibiotics in the world (Garau et al., 2005; European Centre for Disease Prevention and Control [ECDC], 2009; European Surveillance of Antimicrobial Consumption [ESAC], 2009) together with cephalosporins and broad-spectrum penicillins constituting approximately 55% of globally consumed antibiotics (Van Boeckel et al., 2014). Nonetheless, the success of antibiotic therapy is compromised by the development of resistance in many important clinical pathogens (Davies and Davies, 2010). The major cause of resistance to β-lactam antibiotics are β-lactamases, which deactivate antibacterial properties by hydrolysis (Davies, 1994). β-lactamases are found in a broad range of bacteria and include multiple genes that are classified in groups or classes based on functional characteristics and sequence similarities, respectively (Medeiros, 1997; D’Costa et al., 2007). In this context, β-lactamases with similar nucleotide sequences often have similar functional properties, but low sequence similarity is not an indication of different functional properties since genes with low similarity may have similar tertiary structure (Kelly et al., 1982; D’Costa et al., 2007). Furthermore, these genes are often associated with mobile genetic elements (MGEs) that facilitate their transfer within and between bacterial taxa (El Salabi et al., 2013).

Despite evidence that antibiotic resistance (AR) is ubiquitous in non-clinical microbiomes, most studies have assessed the incidence and diversity of β-lactamases in pathogens (Rice, 2001; Paterson and Bonomo, 2005; Ramphal and Ambrose, 2006; Shahid, 2014; Sullivan and Schaus, 2015), and only a few have explored the diversity and distribution of these genes in non-clinical environments. Previous studies that screened β-lactamases in the environment have applied culture-dependent approaches (Villedieu et al., 2003; Gijón et al., 2012; Walsh and Duffy, 2013), culture-independent qPCR-based analyses that target particular antibiotic resistance genes (ARGs) (Pallecchi et al., 2007; Donato et al., 2010; Popowska et al., 2012) and functional metagenomics (Allen et al., 2009; Torres-Cortés et al., 2011; Amos et al., 2014; Forsberg et al., 2014; Su et al., 2014) that provide evidence of β-lactamases activity for novel genes. Recent studies have also applied shotgun metagenomic approaches to assess the scope and abundance of ARGs in natural environments (Yang et al., 2013; Nesme et al., 2014; Li et al., 2015; Fondi et al., 2016). Unlike culture-based methods and functional metagenomics, shotgun metagenomics relies on bioinformatic annotations and therefore, does not provide concrete evidence that β-lactamases are actually active. Nonetheless, this approach provides a global perspective on gene abundance and diversity in targeted microbiomes, at a depth unattainable with the other methods.

Elucidating the global distribution of β-lactamases has great importance. Anthropogenically derived genes can be disseminated to natural ecosystems through sewage, treated wastewater, animal feces, and other sources. Furthermore, β-lactamases have a long evolutionary history in natural environments (Hall and Barlow, 2004; Garau et al., 2005; Aminov, 2009; D’Costa et al., 2011), and there is evidence suggesting that these are reservoirs of novel β-lactamase gene precursors that can be transferred to pathogenic bacteria and contribute to clinically associated AR (D’Costa et al., 2007; Berglund, 2015; Versluis et al., 2015). Despite recent studies that explored resistomes in a range of distinct environments (Bhullar et al., 2012; Cristóbal-Azkarate et al., 2014; Nesme et al., 2014; Czekalski et al., 2015; Fondi et al., 2016), there is a lack of collective knowledge regarding the global diversity and distribution of β-lactamase gene homologs. Although circumstantial evidence implies that β-lactamases have mobilized from natural microbial communities to human-associated microbiomes (Forsberg et al., 2012), the extent of this phenomenon, and the overall dissemination of β-lactamases across ecological and geographic boundaries, is currently unknown.

In this study, we screened 232 metagenomes from ten distinct environments against a collated non-redundant β-lactamase database, and subsequently applied different analyses in order to elucidate the diversity, distribution, and abundance of β-lactamase homologs across environmental and geographic compartments.

## Materials and Methods

### Metagenomic Data Sets

Illumina shotgun metagenomic sequences used in this study were downloaded from two repositories, MG-RAST^1^ and EBI METAGENOMICS^2^, after quality control step performed by each repository. Altogether, we retrieved 232 metagenomes from 27 different projects encompassing 4.7 billion sequences (Supplementary Table S1). The metagenomes were associated with ten environments including soil (non-agricultural and agricultural), freshwater, marine, glaciers, human (from healthy people) and bovine feces, bovine rumen, aerobic and anaerobic compartments of municipal wastewater treatment facilities and anaerobic reactors for industrial food waste digestion.

### Construction of a β-Lactamase Database

A consolidated β-lactamase genes database coined EX-B, integrated four clinically important publically available databases: The Lahey β-lactamase database, The Lactamase Engineering Database (LACED), The Comprehensive Antibiotic Resistance Database (CARD) and The Pasteur Institute’s OXY, OKP, and LEN protein variation databases (Gatica et al., 2016). Sequences deposited into EX-B were aligned using BioEdit 7.2.5 software, generating 1566 non-redundant β-lactamase sequences. The EX-B database is available for download^3^. The short reads generated by Illumina sequencing prevent characterization of specific β-lactamase variants, and therefore we collated variants associated with specific families (i.e., all of the *bla*_OXA_ homologs), resulting in 66 unique β-lactamase gene groups.

### Identification and Characterization of β-Lactamase Gene Homologs in Environmental Metagenomes

Characterization of β-lactamase homologs in the targeted metagenomes was achieved by comparing generated reads to the EX-B database using blastx (Gish and States, 1993), with an identity cutoff greater than 50%, a bit score higher than 30 and an *e*-value lower than 1e^-4^. Although relatively low, we chose this cutoff because environmental β-lactamase homologs can be diverse relative to clinically characterized β-lactamase genes that dominate public databases. The selected cutoff was validated by contrast, using blastx, randomly selected reads coming from different environments against NCBI non-redundant database.

To reduce overlapping results we only registered the best hit for each read. Normalization of sample read abundance enabled comparison of samples with different sequencing depth; and variance analyses (ANOVA with Tukey and non-parametric Wilcoxon tests for unequal number of observations) were performed to identify differences in the relative abundance and β-lactamase gene diversity across the environments analyzed.

We specifically targeted four genes (*bla*_OXA_, *bla*_TEM_, *bla*_CTX-M_, and *bla*_GES_) to determine the level of identity of the reads from the corresponding environments to the EX-B database. Five specific categories were defined based on sequence similarity (50–59%; 60–69%; 70–79%; 80–89%; and 90–100%).

### Ordination of β-Lactamase Genes Assemblages in the Targeted Metagenomes

Identified β-*lactamase* hits from the targeted metagenomes were analyzed in PC-ORD 5.0 (McCune and Mefford, 2011). Initially, the hits were relativized by weighting by ubiquity and transformed by square root. Outliers were identified and removed, and a Bray-Curtis distance matrix using variance regression as an endpoint selection method and Euclidean residual distances to produce scores ranging from 0 to 1 was constructed for use in downstream analysis.

In addition, indicator species analysis was performed in order to pinpoint representative β-lactamases in the targeted environments. This approach is based on a Dufrene and Legendre method that combines information on species abundance and their faithfulness of occurrence in a particular environment. The analysis produces scores from zero (no indication) to 100 (perfect indication). Subsequently, a randomization test (Monte Carlo) based on 4999 runs evaluates the statistical significance of the maximum indicator value recorded for a given species, indicating if the probability to find a given species in a given group is high or low (McCune and Mefford, 2011).

Filtered and normalized hits were analyzed in PC-ORD 5.0 using non-metric multidimensional scaling (NMDS) with a *post hoc* multi-response permutation process (MRPP) to test for similarities, and subsequently with an indicator species analysis to pinpoint β-lactamases in the targeted environments. In addition, Bray-Curtis distance matrices were generated to construct β-lactamase gene networks using EDENetwork 2.18 (Kivelä et al., 2014), after removing β-lactamase hits that could not be characterized to class level. Finally, graphical network visualization and statistical network tests were performed using Cytoscape 3.4.0 (Shannon, 2003).

### Determination of Phylogenetic Distribution in the Targeted Environments

The bacterial community structure of selected environments (agricultural and non-agricultural soils, marine, freshwater, wastewater, human and bovine feces and bovine rumen) was determined by randomly selecting the 16S rRNA phylum-level distributions from 47 metagenomes (Supplementary Table S2), then the average relative abundance per environment was obtained and compared.

## Results

### Richness of β-Lactamases Homologs in the Environment

**Figure 1:**
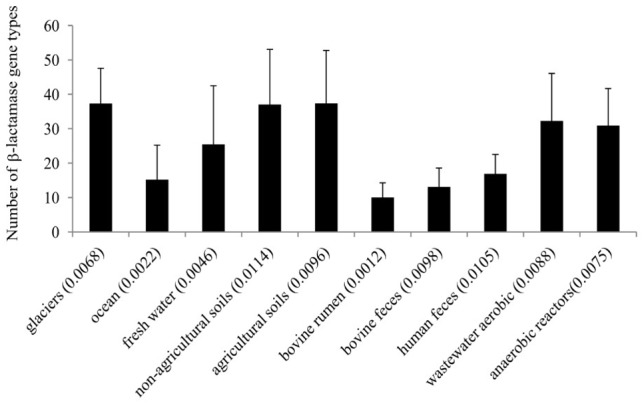
Diversity of β-lactamases across different environments. The average β-lactamase relative abundance in percent of each environment (in brackets) is expressed as the ratio of β-lactamase hits per the total number metagenome reads.

The abundance of β-lactamase gene homologs in the ten targeted environments was on average 0.01% in soil [*n* = 80 metagenomes, standard deviation (STDEV) = 0.002], 0.0068% in glacier (*n* = 10, STDEV = 0.001), 0.0046% in freshwater (*n* = 11, STDEV = 0.001), 0.002% in marine (*n* = 22, STDEV = 0.003), 0.0121 in human feces (*n* = 63, STDEV = 0.007), 0.0049% in bovine (*n* = 27, including feces and rumen samples, overall STDEV = 0.004), and 0.0092% in wastewater treatment plant (*n* = 19), including municipal wastewater (activated sludge and anaerobic digesters) and food waste anaerobic digesters, overall STDEV = 0.003) metagenomes (Supplementary Figure S1 and Supplementary Table S1). Metagenome reads were designated as specific β-lactamase homologs by comparing them to the EX-B database using the blastx algorithm as described in the materials and methods section. Collectively, soil (including agricultural and non-agricultural soils) and glacier metagenomes showed the highest diversity (37 different β-lactamase gene types on average), followed by wastewater metagenomes (30 and 32 different β-lactamase gene types in municipal wastewater treatment facilities and anaerobic digesters, respectively; Figure 1).

**Figure 2:**
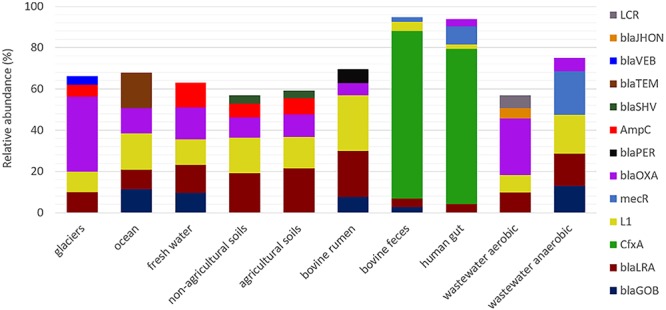
Relative abundance of the five most dominant β-lactamase homologs in the ten environments targeted in this study. Anaerobic reactors encompass both municipal wastewater treatment and food waste treatment digesters.

Analysis of the detected β-lactamase homologs at class level revealed that class A were dominant in bovine and human feces samples (more than 70% in each case); class B were profuse in soils, bovine rumen, marine, and wastewater treatment plants (40–60% in each environment); class C were abundant in soils, fresh water and bovine rumen environments (approx. 20% in each environment); and class D were copious in glaciers, wastewater and freshwater environments (approx. 15–30%; Supplementary Figure S2). The relative abundance of the five most dominant β-lactamase gene homologs in each environment was also assessed (Figure 2). Homologs of *cfxA* were dominant in human and bovine feces, whereas *bla*_OXA_, *bla*_LRA_ and L1 metalo-β-lactamase homologs were dominant in natural environments and wastewater treatment facilities. Overall, there were no differences in distribution of the five dominant β-lactamase homologs in non-agricultural vs. agricultural soils, and between freshwater and soil samples (agricultural and non-agricultural soils) (*p* > 0.05, Wilcoxon test).

Indicator species analysis was applied to pinpoint “core” β-lactamase homologs in selected environments. Four ecosystems with varying inferred levels of “anthropogenic” impact were targeted (human feces, wastewater, agricultural soils, and less anthropogenically impacted environments, which included non-agricultural soils, glaciers, marine, and freshwater samples). There was an inverse relation between the extent of “anthropogenicity,” and the number of indicator species observed. In total, 25 β-lactamase family homologs showed a high probability of occurrence in the less anthropogenically impacted environments, and only four (*bla*_EBR_ *cfxA*, HGI, and *mec*R) exhibited a high probability of occurrence in the human fecal samples (Table 1).

**Table 1:** Faithfulness of β-lactamase homolog occurrence based on indicator species analysis in selected metagenomes with varying levels of anthropogenic impact.

Environment	High probability of β -lactamase homolog
Human feces	*bla*_EBR_, *cfxA*, HGI, *mec*R
Wastewater treatment plants	*bla*1, *bla*_FEZ_, *bla*_GOB_, *bla*_NPS_, *bla*_SIM_, *bla*_TEM_, *bla*_TOHO_, *bla*Z, blm, LCR
Agricultural soils	*Amp*C, *bla*_ACT_, *bla*_B_, *bla*_LEN_, *bla*_LRA_, *bla*_MOX_, *bla*_NDM_, *bla*_OCH_, *bla*_SHV_, *bla*_V IM_, *Cep*S, THINB
Less anthropogenically impacted (include non-agricultural soils, glaciers, marine, and fresh water samples)	*bla*_ACC_, *bla*_CARB_, *bla*_CMY_, *bla*_CTX-M_, *bla*_DHA_, *bla*_FONA_, *bla*_GES_, *bla*_IND_, *bla*_JOHN_, *bla*_KHM_, *bla*_KLUA_, *bla*_KPC_, *bla*_MIR_, *bla*_OKP_, *bla*_OXA_, *bla*_OXY_, *bla*_PDC_, *bla*_PER_, *bla*_SME_, *bla*_SPM_, *bla*_TUS_, *bla*_V EB_, C*ph*A, L1, SRT

### Inter- and Intra-Environmental β-Lactamase Associations

**Figure 3:**
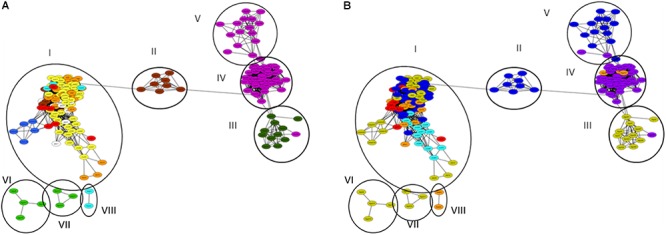
β-lactamase homologs gene network of analyzed metagenomes. Circles and Roman numerals represent clusters defined using network analyzer in Cytoscape. **(A)** Network color-code based on environment: bovine rumen (light green); bovine feces (dark green); human feces (purple); aerobic (activated sludge) municipal wastewater treatment (red), anaerobic municipal waste and food digestion reactors (brown); non-agricultural soil (yellow); agricultural soil (orange); glaciers (white); fresh water (light blue); marine (dark blue). Anaerobic municipal waste samples were primarily associated with Cluster I, whereas anaerobic food digesters all grouped in Cluster II. **(B)** Network color-coded based on geographical origin: United States (blue); Israel (light blue); Northern Europe (purple), Central Europe (orange); China (red); Australia (yellow).

We constructed distance matrices and gene networks from all of the metagenome derived β-lactamase hits, and exclusively from well-defined hits characterized as specific β-lactamase families (Supplementary Figure S3), this means that hits defined only as β-lactamase or class β-lactamase were discarded from analysis. In both cases, the topography of the generated β-lactamase gene networks formed eight clusters, with a high level of connectivity within the individual clusters, but with low connectivity between the clusters (Supplementary Tables S3, S4). Cluster I collated all of the soil, freshwater, marine, glacier, and municipal wastewater metagenome-derived β-lactamase homologs; cluster II harbored the food waste anaerobic digester samples; cluster III contained the bovine feces samples; cluster IV and V contained human feces samples (from Europe and the United States, respectively); cluster VI and VII contained bovine rumen samples; and cluster VIII contained freshwater samples. Color coding of the metagenomes by both environmental (Figure 3A) and geographic (Figure 3B) origin revealed high connectivity between metagenomes from similar environments, and limited or no connectivity between samples defined by geographic origin. In some cases such as in the human feces samples, geographic influence occurred within individual environments. Cluster IV encompassed human feces metagenomes from Northern and Central Europe (*n* = 44), whereas closely associated cluster V contained all of the metagenomes from the United States (*n* = 15). This geographic differentiation should, however, be taken with caution considering the potential impact of additional differentiating factors such as age and gender. In order to highlight potential interactions between β-lactamase homologs from the natural ecosystems encompassed in Cluster I, we applied several network indices including closeness centrality, radiality, topological coefficients, neighborhood connectivity, and number of directed edges (Supplementary Tables S5, S6). In general, Cluster I exhibited high values of several measures of network centrality confirming the close relationship of the β-lactamase homologs in these environments. Specifically, most of the non-agricultural soil metagenomes (und_1, 4, 12, 22, 25, 27, 28, 30, 31, 55, and 56) were centrally situated in this cluster and exhibited the highest level of connectivity along with a glacier sample (glac_2), and two freshwater samples (fresh_8 and 17). In contrast, connectivity was generally lower in the agricultural soil samples, and these were less centrally situated within the cluster. Evidence suggested that the observed divergence in the agricultural soil samples was strongly associated with plant species, but this could not be statistically validated.

We specifically assessed the microbiomes (at phylum level based on 16S rRNA gene analysis) of eight selected environments (Supplementary Figure S4) to determine potential correlations between β-lactamase composition and bacterial community structure. Collectively, this phylogenetic characterization revealed two groups. “Anaerobic environments,” encompassing human and animal feces and rumen samples that contained high abundances of *Firmicutes* (relative abundance of 33.6–50.4%) and *Bacteroidetes* (19.7–24.7%); and “aerobic environments,” dominated by *Proteobacteria* (22.7–49.4%). Based on this classification, the diversity of β-lactamases was lower in anaerobic environments (although this was not always statistically significant), but the β-lactamase richness across environments did not show differences between the aerobic and anaerobic environments.

### Similarity of Environmental β-Lactamase Homologs to Clinical-Associated β-Lactamases

**Figure 4:**
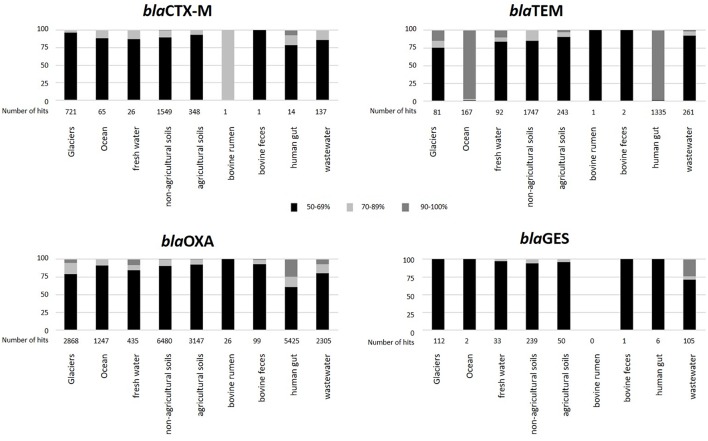
Relative abundance of selected β-lactamases in the targeted environments grouped according to identity (%) to EX-B database sequences. Black: 50–59%; gray: 60–69%; white: 70–79%; white granulated: 80–89%; and black granulated: 90–100%.

To elucidate the level of similarity between environmental homologs and β-lactamase genes from pathogenic bacteria, we specifically targeted four prevalent clinically associated genes: *bla*_CTX-M_, *bla*_GES_, *bla*_OXA_, and *bla*_TEM_, in the metagenomes of the targeted environments. This was based on the notion that the β-lactamase sequences in the EX-B database used in this analysis are predominantly associated with clinically derived bacteria. Generated BLAST hits were grouped in five categories according to the level of identity of the environmental β-lactamase homologs to the database sequences (50–59%; 60–69%; 70–79%; 80–89%; and 90–100%; Figure 4). For the most part, the natural environments showed relatively low levels of homology to the four β-lactamases. In contrast, β-lactamase homologs from municipal wastewater treatment plants, anaerobic food and wastewater digesters and human feces metagenomes displayed higher levels of similarity, suggesting that these might be more “anthropogenically impacted.” Although *bla*_OXA_ was the most abundant β-lactamase in all samples, *bla*_TEM_ showed higher levels of similarity to the database sequences, especially in the marine and human feces metagenomes. Interestingly, almost all of the highly similar (90–100%) *bla*_TEM_ hits in the human feces were related to one specific metagenome (H_gut 8, 1196 hits), suggesting that this sample may be outside the norm and potentially associated with dysbiosis due to antibiotic therapy. Collectively, most of the hits from all of the targeted environments showed less than 70% homology to these four targeted β-lactamases, although high levels of database identity (between 90 and 100%) were observed in some of the targeted environments such as human feces (*bla*_TEM_ and *bla*_OXA_), oceans (*bla*_TEM_), and wastewater (*bla*_GES_) metagenomes.

**Figure 5:**
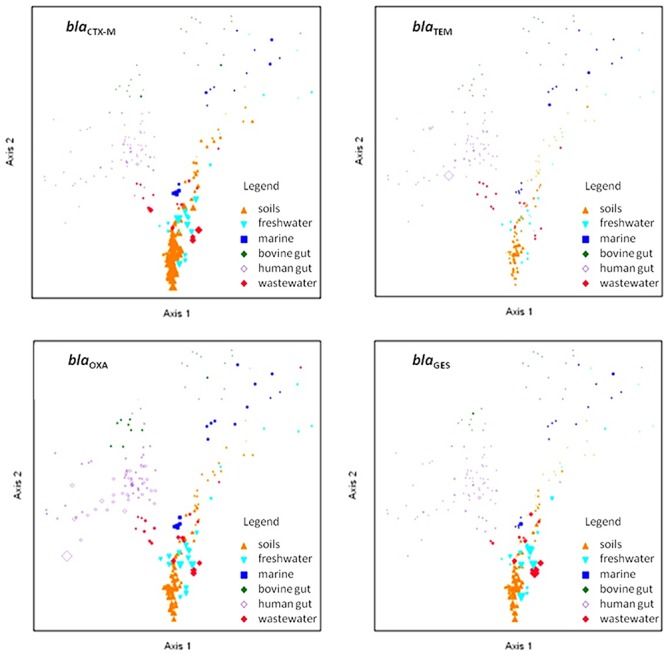
Non-metric multidimensional scaling ordination of selected β-lactamase gene homologs. Shapes and colors represent the environmental source of the metagenome samples. The point sizes are proportional to the abundance of the depicted gene. The PC-ORD generated stress was between 15.2 and 15.5, depending on the number of iterations and dimensions applied.

We plotted and visualized the abundance and distribution of the four selected β-lactamase genes across the targeted environments using NMDS ordination (Figure 5). Homologs associated with *bla*_OXA_ were the most widely distributed, with ubiquitous presence in soil, fresh water, marine, and human feces metagenomes. Conversely, *bla*_CTX-M_ homologs were less abundant, and primarily identified in soils, while *bla*_GES_ homologs were profuse in soils and in wastewater environments. Interestingly, despite the copiousness of *bla*_TEM_ in clinical environments, it was not highly distributed across the analyzed environments.

Finally, to support the application of the 50% similarity cutoff, we applied the blastx algorithm to randomly selected reads (*n* = 40) coming from different environments analyzed in this study, against the NCBI non-redundant gene database (Supplementary Table S7). The results indicated that in only five instances, the first hit was not a β-lactamase, and of these, only one query was not characterized as a β-lactamase in the first ten hits obtained. In general, the EX-B database was more restrictive than NCBI database in 31 cases.

## Discussion

Understanding ARG dynamics on a global scale is highly complex. As stipulated in the resistome hypothesis (D’Costa et al., 2006), the long evolutionary history of ARGs makes soil and other natural ecosystems highly diverse reservoirs of ARG precursors that can potentially be transferred to human pathogens (Hall and Barlow, 2004; Garau et al., 2005; Baltz, 2008; Aminov, 2009). Concurrently, dissemination of ARGs from anthropogenic sources to natural environments (Czekalski et al., 2015) can affect the structure of these natural resistomes and contribute to the global scope of AR. In order to enhance understanding of the environmental dimensions of AR and elucidate relationships between natural and anthropogenically associated resistomes, we evaluated the scope, diversity and distribution of β-lactamase gene homologs on a global scale by targeting a multitude of shotgun metagenomes from ten distinct environments. We applied relatively modest cutoff criteria (identity ≥ 50%; bit score ≥ 30; *e*-value ≤ 1e^-4^) for defining specific β-lactamases. This is lower than some previously conducted studies (i.e., Yang et al., 2016 used identity ≥ 60%; *e*-value ≤ 1e^-7^), but similar to other studies that used lower cutoffs to pinpoint ARGs in metagenomics reads (i.e., Nesme et al., 2014 used identity ≥ 35%; bit score ≥ 60). Application of lower stringency may capture many β-lactamase gene homologs such as esterases (Wagner et al., 2002), proteases (Delfosse et al., 2009), and transpeptidases (Massova and Mobashery, 1998); and therefore, our analyses cannot determine that the pinpointed homologs are actually functional β-lactamases. Conversely, the relation between environmental ARG precursors and homologous clinical genes that dominate public databases is often low (Forsberg et al., 2014) and therefore, by applying highly stringent cutoffs, we may omit a substantial fraction of the environmental resistome. The selected cutoff was validated by contrast, using blastx, randomly selected reads against NCBI non-redundant database; thus, the results indicated that even when a low similarity was observed (50%), the reads were more close to ARGs than to any other type of gene. Even more, usually, reads contrasted against NCBI database, presented a higher percent of identity to β-lactamases than the observed using EX-B database.

The β-lactamase homologs pinpointed in this study distinctly grouped into four mega-clusters (human and bovine feces, bovine rumen, anaerobic food waste digesters, and natural environments that included both aerobic and anaerobic municipal wastewater treatment plant samples). These clusters were primarily molded by ecology and not geography, similar to results previously reported (Fondi et al., 2016). The bovine and human feces β-lactamase clusters shared relatively high levels of connectivity, suggesting exchange of ARGs between these environments (via transfer of bacteria or horizontal gene transfer). Several previously published reports describing β-lactamase genes in bacteria from food-producing animals (Hasman et al., 2005; Cloeckaert et al., 2007; Smet et al., 2008; Costa et al., 2009), companion animals (Costa et al., 2004; Carattoli et al., 2005; Moreno et al., 2008), and wild animals (Costa et al., 2006; Poeta et al., 2008) with high similarity to β-lactamases from clinically associated microbiomes support this phenomenon. Four β-lactamase genes (*bla*_EBR_, *cfxA*, *mecA HGI*) previously identified in human-associated pathogenic and commensal Gram-positive and Gram-negative bacteria (Bellais et al., 2002; Avelar et al., 2003; Ferreira et al., 2007; Sommer et al., 2009; Hu et al., 2013; Ballhausen et al., 2014) in hospital and municipal wastewater (Schwartz et al., 2003; Bockelmann et al., 2008), were ubiquitous to these environments based on indicator analyses. In both human and bovine feces, *cfxA* homologs dominated the β-lactamase gene pool, indicating a selective advantage of this gene in the mammal feces. This gene is ubiquitous to *Bacteroidetes* isolates from anaerobic human-associated environments (Meggese and Abratt, 2015; Binta and Patel, 2016), which as describe above was highly copious in both the human and bovine feces analyzed in this study. The fact that *cfxA*, is frequently associated with the Tn4555 transposon (Agga et al., 2015), indicates its capacity to be horizontally transferred between phylogenetically related strains. This suggests that when evaluating microbial transfer between animals and humans, specific ARGs should be monitored concurrent to evaluation of zoonotic pathogens.

In contrast to the relatively strong interactions observed between the bovine and human fecal metagenomes, almost no network connectivity occurred between either of the fecal clusters and “environmental” cluster I. These findings are in accordance with metagenomics (Agga et al., 2015; Li et al., 2015; Munck et al., 2015; Fondi et al., 2016) and more classical approaches (Smillie et al., 2011; Forsberg et al., 2014), which reported specificity of ARGs to particular environments, with a high degree of intra-habitat mobility and low probability of ARG transfer between different environments. The significant differences in bacterial community composition between the “fecal” and “environmental” samples suggests that horizontal transfer of ARGs is often constrained by phylogenetic boundaries as previously indicated for the soil microbiome (Forsberg et al., 2014). Furthermore, other factors such as founder effects, ecological connectivity between donor and acceptor, fitness costs or second-order selection may limit the gene transfer of ARGs between environments (Martinez, 2012; Perry and Wright, 2013; Forsberg et al., 2014).

Specific evaluation of *bla*_TEM_, *bla*_CTX-M_, *bla*_GES_, and *bla*_OXA_ (β-lactamases characteristic of certain Gram-negative pathogens) in the targeted environments, revealed high abundance of *bla*_CTX-M_ in soils, and ubiquitous distribution of *bla*_OXA_ genes across all of the analyzed environments. The similarity between the environmental β-lactamases and the clinically derived database sequences was generally low (50–60%), supporting the hypothesis that natural environments may be reservoirs of precursor ARGs that can eventually emerge in human pathogens (D’Costa et al., 2006). Conversely, these low levels of identity also indicate that pathogen-associated β-lactamases are not profuse in natural environments. Albeit the resistome hypothesis (D’Costa et al., 2007), the low connectivity observed between natural β-lactamase clusters and fecal clusters in the network analysis suggest that these events occur at relatively low frequencies. This is supported by several studies (Riesenfeld et al., 2004; Forsberg et al., 2014; Hatosy and Martiny, 2015), which also suggest limited mobility of ARGs between environmental and human-associated microbiomes. This analysis targeted a limited scope of clinically important β-lactamases, and undoubtedly, analysis of other important genes such as *bla*_SHV_ need be performed in the future.

The findings presented here provide a holistic overview of β-lactamase homologs across a diverse array of environments. Nonetheless, the results should be taken with caution because screening of metagenomic data only revealed the most dominant β-lactamases in the targeted environments, and therefore evidence of gene transfer for less abundant genes will inevitably be overlooked using this approach. Furthermore, data from public metagenome projects is often highly heterogeneous, and although all of the targeted metagenomes analyzed in this study used Illumina technology, each project employed different methodologies and protocols, which may influence the obtained results. For instance, the marine metagenomes from the Sydney Harbor project (Supplementary Table S1) were sampled at 0.3 m depth, whereas the metagenomes from the Amazon continuum project were obtained from 3.63 to 4.47 m depth, at different distances from the coast. This may cause substantial differences in acquired resistomes, based on previous studies that have shown differences in ARG profiles at different water depths (Mudryk and Skórczewski, 2000; Di Cesare et al., 2015). In addition, agronomic practices can significantly affect the resistomes of agricultural soils as implied in a recent study that demonstrated that manure application stimulates shifts in soil bacterial community and ARG composition (Agga et al., 2015). In the case of feces metagenomes, the health condition and medicated status of the sampled individuals can clearly affect both the microbiological and genomic content. While ubiquity, power transformations and outlier removal partially reduces these effects, the lack of metadata associated with metagenomes can substantially complicate data interpretation.

Collectively, this study clearly indicates that both ecology and phylogeny are major driving forces in determining β-lactamase distribution in the environment, and that natural ecosystems are important reservoirs of β-lactamase gene homologs. Despite the potential exchange of β-lactamases within individual environments and between natural ecosystems and wastewater facilities, our results suggests that the exchange of β-lactamases between natural environments and human and bovine fecal microbiomes occurs at low frequencies, and therefore these events are difficult to detect. Finally, our results supports previous studies that suggest limited mobility of ARGs from natural to human-associated environments. Notwithstanding the above, metagenomics analyses only pinpoint profuse genes and we cannot rule out the notion that rare events may facilitate the transfer of novel β-lactamases from natural environments to human/animal microbiomes, thereby contributing to the propagation of AR. Future studies should focus on pinpointing these events and assessing the epidemiological risks associated with them.

## Author Contributions

JG drafted the main manuscript and performed the data analysis. EJ and EC were responsible for guiding and supporting the experiment and manuscript revisions.

## Conflict of Interest Statement

The authors declare that the research was conducted in the absence of any commercial or financial relationships that could be construed as a potential conflict of interest.

## Footnotes

1 http://metagenomics.anl.gov/

2 https://www.ebi.ac.uk/metagenomics/

3 http://app.agri.gov.il/eddie/EX-B_Database/EX-B_Database.fasta
