# Supplementary material for: Comparative Metagenomics and Network Analyses Provide Novel Insights Into the Scope and Distribution of β-Lactamase Homologs in the Environment
Source: Front Microbiol. 2019 Feb 11;10:146. doi: 10.3389/fmicb.2019.00146 (PMC6378392; doi:10.3389/fmicb.2019.00146)
Supplement: Supplementary file 1 [file Data_Sheet_1.PDF]

Supplementary Material

Comparative metagenomics and network analyses provide novel insights into the scope and distribution of β-lactamases homologues in the environment.

Joao Gatica[1,2], Edouard Jurkevitch[3] and Eddie Cytryn[1*]

1 The Institute of Soil, Water and Environmental Sciences, The Volcani Center, Agricultural Research Organization, Rishon Lezion, Israel.

2 The Department of Soil and Water Sciences, The Robert H. Smith Faculty of Agriculture, Food and Environment, The Hebrew University of Jerusalem, Rehovot, Israel.

3 The Department of Plant Pathology and Microbiology, The Robert H. Smith Faculty of Agriculture, Food and Environment, The Hebrew University of Jerusalem, Rehovot, Israel.

*** Correspondence to** eddie@volcani.agri.gov.il

1. Supplementary figures and tables
1.1 Figures

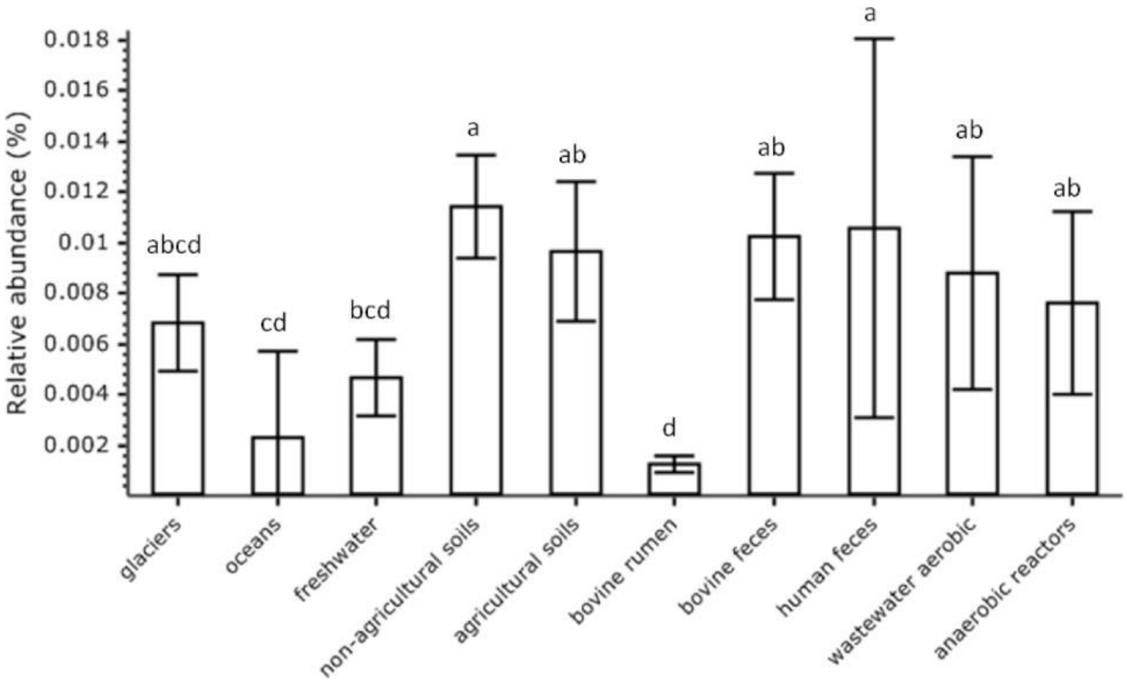

Figure S1. Relative abundance of β-lactamase homologues detected in shotgun metagenomes from different environments targeted in this study. Similarity in the diversity of β-lactamase homologue genes was tested by ANOVA and is represented by letters. Anaerobic reactors encompass both municipal wastewater treatment and food waste treatment digesters.

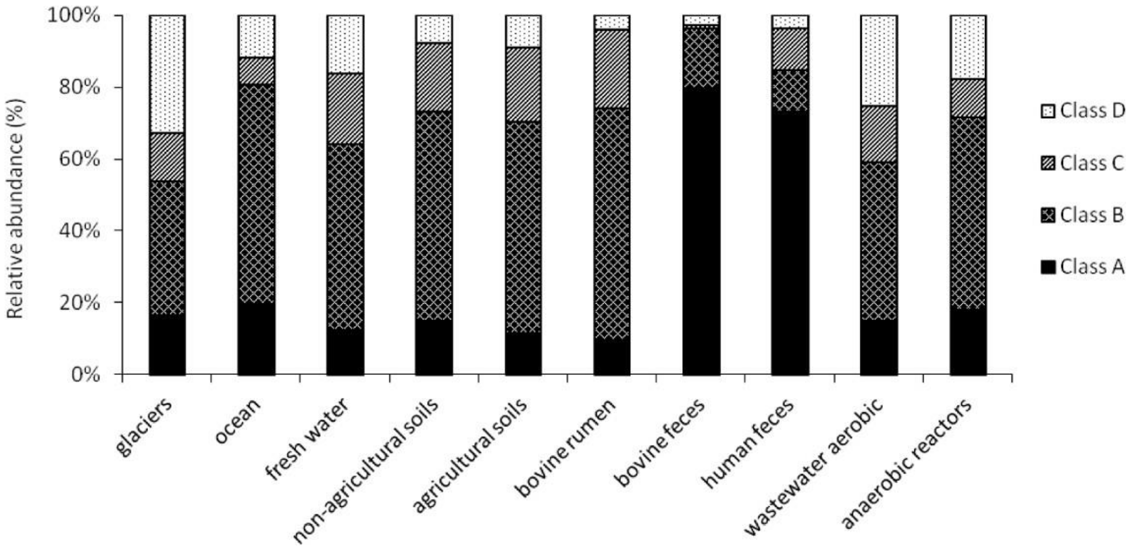

Fig. S2. Relative abundance of β-lactamase molecular classes in the targeted environments.

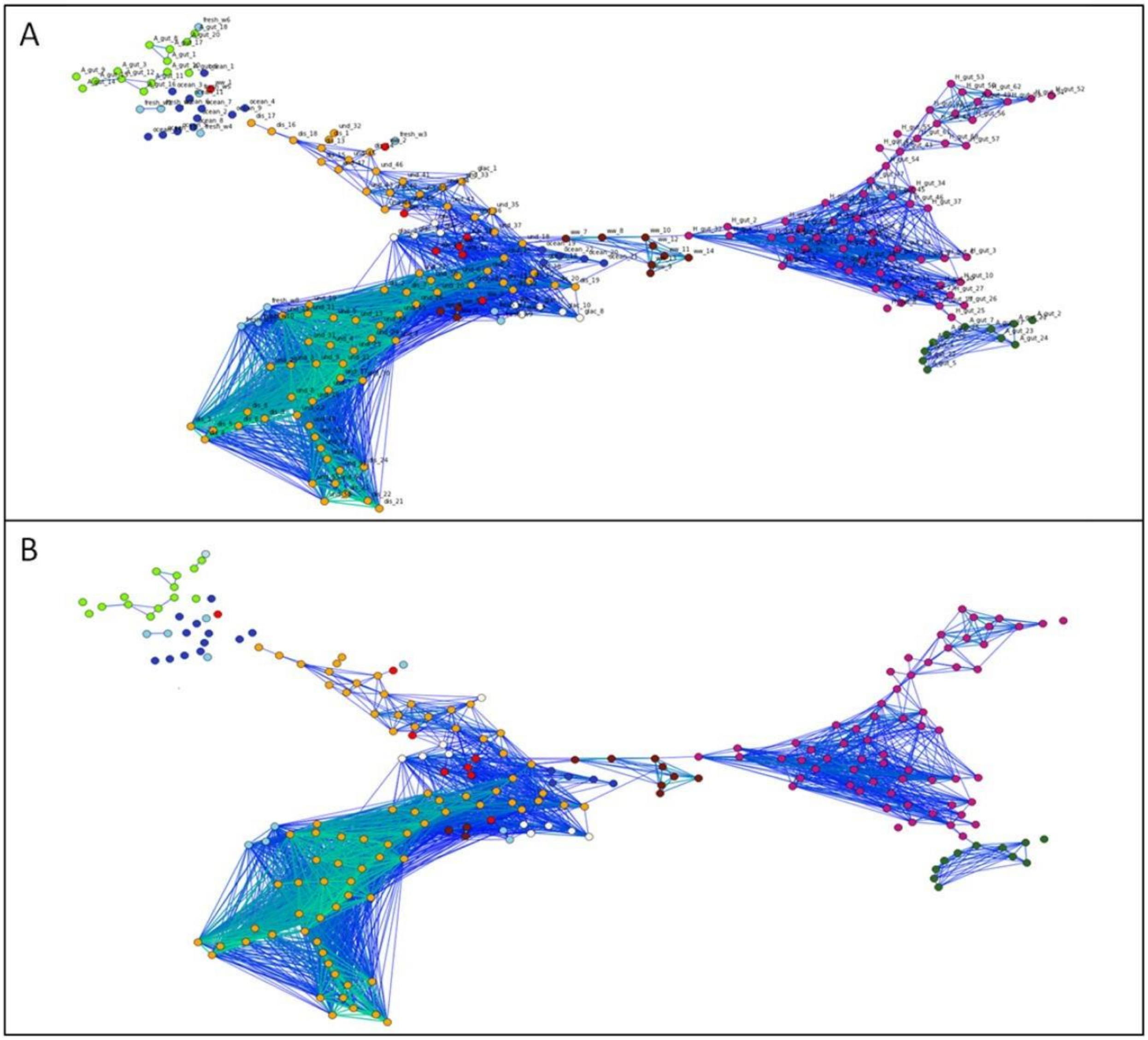

Fig. S3. β-lactamase gene network of analyzed metagenomes, including all blast hits (i.e. hits defined at gene level and hits defined at class level). (**A**) Network with sample names and color-code based on environment: bovine rumen (light green); bovine feces (dark green); human feces (purple); municipal wastewater (red), anaerobic digester (dark red); soils (agricultural and non-agricultural soils) (orange); glaciers (white); fresh water (light blue); marine (dark blue). (**B**) Network color-code based on environment (legend above).

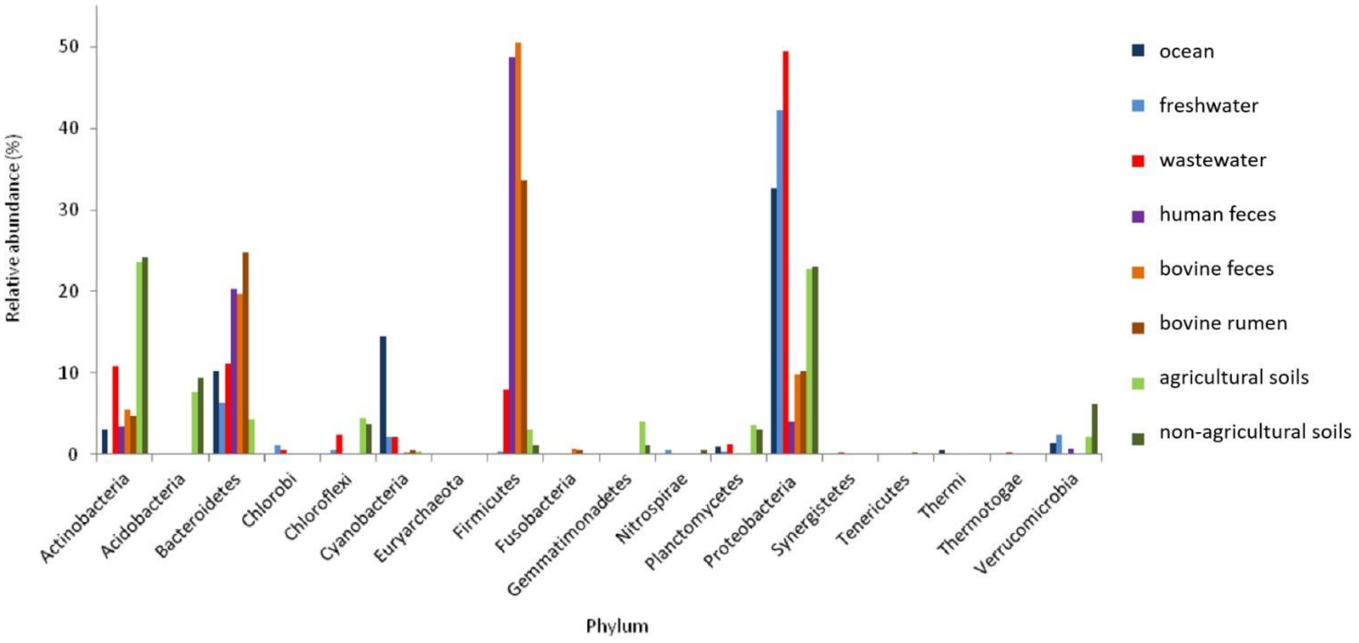

Fig. S4. Phylogenetic composition (in average) of analyzed environments at phylum level. Legend: dark blue: marine metagenomes, light blue: freshwater; red: wastewater; human feces: purple; bovine feces: orange; bovine rumen brown; agricultural soils: light green; non-agricultural soils: dark green.

1.2 Tables

Table S1. Metagenomes analyzed in this study and metadata related to each metagenome.

Project	MGRAST/EBI ID	ID in our study	Number of reads	N of reads after QC	β-lactamase hits	% β-lactamases of total high quality reads	Biome	Feature	Country	Location	Contact
**Reconstructing the microbial diversity and function of pre-agricultural tallgrass prairie soils in the United States** https://www.ebi.ac.uk/metagenomics/projects/ERP003954	ERS351525	und_1	27026728	26781100	3537	0.013207	Temperate grassland	soil	USA	Iowa	Noah Fierer
	ERS351524	und_2	17269151	17169406	1726	0.010053				Minnesota	
	ERS351523	und_3	37838514	37619975	4819	0.01281				Kansas	
	ERS351522	und_4	28408245	27168429	3204	0.011793				Oklahoma	
	ERS351521	und_5	28965564	28613189	2885	0.010083				Nebraska	
	ERS351520	und_6	14783360	14642447	1825	0.012464				Minnesota	
	ERS351519	und_7	29165527	28956367	3817	0.013182				Iowa	
	ERS351518	und_8	35577828	35367944	4059	0.011476				Oklahoma	
	ERS351517	und_9	28645040	28462402	3974	0.013962				Minnesota	
	ERS351516	und_10	18124186	18024134	2119	0.011756					
	ERS351515	und_11	21210826	21041722	2841	0.013502				South Dakota	
	ERS351514	und_12	21949310	21766496	3017	0.013861				Nebraska	
	ERS351513	und_13	21683990	21464055	2800	0.013045				Nebraska	
	ERS351512	und_14	21989672	21876140	2847	0.013014				Minnesota	
	ERS351511	und_15	18351280	18243784	2463	0.0135					
	ERS351510	und_16	29082154	28817452	3798	0.01318				Iowa	
	ERS351509	und_17	28546560	28403061	3830	0.013484				Nebraska	
	ERS351508	und_18	12254776	12009305	1197	0.009967				Minnesota	
	ERS351507	und_19	23293397	23061470	2707	0.011738				Texas	
	ERS351506	und_20	18809837	16692337	2212	0.013252				South Dakota	
	ERS351505	und_21	29543534	29356512	3900	0.013285				Nebraska	
	ERS351504	und_22	31952130	30859688	4005	0.012978				Texas	
	ERS351503	und_23	25936857	25714694	3432	0.013346				Iowa	
	ERS351502	und_24	28422926	25909532	3228	0.012459				Kansas	
	ERS351501	und_25	15171524	15093073	1670	0.011065				South Dakota	
	ERS351500	und_26	18850947	18737555	2470	0.013182				Minnesota	

	ERS351499	und_27	16531308	16431017	2174	0.013231					
	ERS351498	und_28	13692283	13569870	1805	0.013302				North Dakota	
	ERS351497	und_29	41473633	41112030	5426	0.013198				Nebraska	
	ERS351496	und_30	28434992	28276297	3546	0.012541				South Dakota	
	ERS351495	und_31	22571884	22409684	2993	0.013356				Nebraska	
**GED (Great Prairie Iowa assembly)**	4504798	und_32	3096464	3096463	240	0.0078	terrestrial habitat	soil	USA	Iowa	C. Titus Brown
	4504797	dis_1	1847717	1847717	197	0.0107					
**Comparative metagenomic analysis of soil microbial communities varying in disease suppression potential** https://www.ebi.ac.uk/metagenomics/projects/ERP004492	ERS407417	dis_2	28741036	19768283	2299	0.01163	Cropland	soil	Australia	Kallora	Paul Greenfield Paul Greenfield
	ERS407418	dis_3	41909110	29380094	3749	0.01276					
	ERS407419	dis_4	39237625	27603190	3392	0.012288					
	ERS407420	dis_5	39454220	28498986	3500	0.012281					
	ERS407421	dis_6	37448206	27155014	3605	0.013276					
	ERS407422	dis_7	22325386	16196817	1948	0.012027					
	ERS407423	dis_8	50321072	35256190	4503	0.012772					
	ERS407424	dis_9	45195994	32658408	1726	0.005285					
**EarlhamMetagenomes_2012** metagenomics.anl.gov/metagenomics.cgi?page=MetagenomeProject&project=2850	4508938	dis_10	9999775	9689648	1258	0.01258	Agricultural soil	soil	USA	Indiana	Chris Smith
	4508939	dis_11	16923988	16395206	1795	0.010948					
	4508940	dis_12	17442824	17014984	1878	0.011037					
**Cross-site soil metagen** metagenomics.anl.gov/metagenomics.cgi?page=MetagenomeProject&project=2997	4477803	und_33	5951684	5818728	452	0.007768	shrubland biome		USA	Antarctica	Noah Fierer
	4477805	und_34	5899497	5856032	476	0.008128				Mojave desert (California)	
	4477875	und_35	5235352	5176612	674	0.01302	moist broadleaf forest			New Mexico	
	4477877	und_36	6402940	6248585	795	0.012723	coniferous forest			Alaska	
	4477904	und_37	10863646	10683655	779	0.007292	shrubland biome			Antarctica	
**Soil microbiome analysis along a precipitation gradient**	4654021	und_38	9681819	9640724	1023	0.010611	bulk soil	soil	Israel	Galilee mountains	**Binu Tripathi**
	4654022	und_39	4484452	4466739	489	0.010948					
	4654023	und_40	3762792	3746657	427	0.011397				Terra rossa	
	4654025	und_41	3808872	3791416	425	0.01121					

	4654026	und_42	4902736	4878882	620	0.012708				Rendzina	
	4654027	und_43	2840653	2824195	405	0.01434					
	4654028	und_44	3129422	3090942	360	0.011647					
	4654029	und_45	2725118	2712235	225	0.008296				Negev plateau	
	4654030	und_46	2915911	2903190	295	0.010161					
	4654031	und_47	2528610	2516149	239	0.009499					
	4654032	und_48	4053435	4034735	476	0.011798				Galilee mountains	
**Microbial community of mobilong acid sulfate soil depth profile using metagenomes** https://www.ebi.ac.uk/metagenomics/projects/ERP005307	ERR476938	dis_13	3653926	3372138	233	0.00691	grassland	soil	Australia	Mobilong	Xiaoyi Wang
	ERR476939-40	dis_14	4765237	4316863	269	0.006231					
	ERR476941	dis_15	3559506	3160182	176	0.005569					
	ERR476942	dis_16	3567229	3221991	182	0.005649					
	ERR476943	dis_17	3607779	3194026	153	0.00479					
	ERR476944-45-46	dis_18	5059527	4542175	315	0.006935					
**Soil metagenome sampled from a chitin-amendment agricultural field**	ERS950673	dis_19	13734269	13351896	1222	0.009152	bulk soil	soil	UK	Kilkeel	Ian Lidbury
	ERS950674	dis_20	13734269	12898341	1169	0.009063					
**Biomes of australian soils environmnets**	ERR671910	dis_21	89007697	75263225	6929	0.009206	agricultural soil	soil	Australia		Anna Fitzgerald
	ERR671913	dis_22	74681700	63439290	6984	0.011009					
	ERR671915	und_49	75473349	50523311	4551	0.009008	woodland			Victorian Mallee-Walpeup	
	ERR671916	und_50	86680096	72233421	7434	0.010292					
	ERR671919	dis_23	86588959	73359053	6680	0.009106	agricultural soil				
	ERR671921	dis_24	86227789	56724124	5788	0.010204					
	ERR671922	und_51	96898846	82844947	6838	0.008254	grassland			Uluru	
	ERR671927	und_52	82179459	69344940	6466	0.009324					
	ERR671931	und_53	75407538	61807045	5015	0.008114					
	ERR671932	und_54	87192508	68431179	5586	0.008163					
	ERR671934	und_55	91347085	78285600	6526	0.008336					
	ERR671938	und_56	83136657	65338948	5460	0.008356					
**Rotmoosferner glacier cryoconite metagenome**	4491734	glac_1	9727829	9679520	359	0.003709	Polar domain	glacial	Austria	glacial	Arwyn Edwards

**Light-dependent microbial metabolisms drive carbon fluxes on glacier surfaces**	ERS1030529	glac_2	26366877	25260533	1379	0.005459	Polar domain	glacial	Italy	glacial	Andrea Franzetti
	ERS1030531	glac_3	24706158	23783017	1379	0.005798					
	ERS1030532	glac_4	23641277	22753034	1100	0.004835					
	ERS1030533	glac_5	12617681	12058114	747	0.006195					
	ERS1030534	glac_6	23163525	22180707	2126	0.009585					
	ERS1030535	glac_7	21253629	20421120	1913	0.009368					
	ERS1030536	glac_8	16143554	15493519	1142	0.007371					
	ERS1030537	glac_9	21497679	20712670	1615	0.007797					
	ERS1030539	glac_10	17279598	16584142	1320	0.007959					
**Metagenomics of a freshwater pond in Sheffield, UK**	ERR732063	fresh_w1	2371867	2222846	81	0.003644	fresh pond	water	UK	Sheffield	Jags Pandhal
	ERR732064	fresh_w2	2371867	2211332	97	0.004386					
**Amazon Continuum Metagenomes** *samples taken at diff depth and diff location	SRS565747	ocean_1	5558497	4028446	32	0.000794	ocean	seawater	Western Tropical North Atlantic Ocean		
	SRS565748	ocean_2	8051114	5732187	89	0.001553					
	SRS582461	ocean_3	6477817	4706714	92	0.001955					
	SRS584254	ocean_4	6957336	4805124	146	0.003038					
	SRS565994	ocean_5	4812849	3439683	50	0.001454					
	SRS565995	ocean_6	8271106	4348396	108	0.002484					
	SRS567311	ocean_7	7880865	7179963	109	0.001518					
	SRS581964	ocean_8	7979669	6905208	73	0.001057					
	SRS581965	ocean_9	10978721	9150070	170	0.001858					
	SRS582462	ocean_10	3683906	3057834	12	0.000392					
	SRS577849	ocean_11	4858345	4284498	63	0.00147					
	SRS580495	ocean_12	4634087	3643413	627	0.017209					
	SRS580498	ocean_13	4609128	4034489	19	0.000471					
	SRS582491	ocean_14	4254694	3689692	65	0.001762					
	SRS584253	ocean_15	6676513	6115467	35	0.000572					
	SRS580500	ocean_16	4708619	3530780	31	0.000878					
	SRS580502	ocean_17	3520315	2795546	13	0.000465					
	ERR1198911	fresh_w3	2817782	2525463	207	0.008197	cave	water	Romania	Mangalia	

**Microbial diversity in a cave system**	ERR1198912	fresh_w4	1195020	1054515	63	0.005974					Deepak Kumaresan
	ERR1198913	fresh_w5	1401432	1277939	51	0.003991		sediment			
	ERR1198914	fresh_w6	1154044	1044050	38	0.00364					
**Coupled metagenomic and metatranscriptomic study of the Columbia river coastal margin saliniti gradient**	Err864071	fresh_w7	77687621	41537224	2222	0.005349	fresh and marine waters	water	USA	Columbia river	Caroline Fortunato
	Err864069	fresh_w8	91390537	64083903	3372	0.005262					
	Err864077	fresh_w9	134043614	51586330	1524	0.002954					
	Err864073	fresh_w10	109957314	91329495	3015	0.003301					
	Err864075	fresh_w11	106153626	86255432	3794	0.004399					
**Sydney Harbour metagenomes**	ERR514703	ocean_18	59964677	44231900	843	0.001906	aquatic biome	seawater	Australia	Sydney	Denis O'Meally
	ERR514704	ocean_19	63122676	46423406	1032	0.002223					
	ERR514705	ocean_20	65537143	47570603	1112	0.002338					
	ERR514706	ocean_21	62496982	45462913	1260	0.002771					
	ERR514707	ocean_22	60379599	46205288	1043	0.002257					
**MiDAS-DK**	4611649	ww_1	285799	283960	52	0.018312	activated sludge	wastewater	Denmark	Aalborg	Mads Albersten
**HKULG209_1**	4511202	ww_2	10737272	10219844	246	0.002407	waste water	wastewater	China	Shangai	Tong Zhang
**Stanley WWTP**	4511202	ww_3	16663946	16287845	1823	0.011192	activated sludge	wastewater	China	Hong Kong	Tong Zhang
**Anaerobic-anoxic sludge community**	4521534	ww_4	17595000	16861433	2367	0.014038	sludge	wastewater	China	Shenzhen xili	Xiaomei lv
	4522552	ww_5	17595000	16861433	2172	0.012881					
	4524971	ww_6	16897424	16020903	2022	0.012621					
**Beer to caproate**	4480719	WW_7	27029730	25097245	1559	0.006212	anaerobic sludge	wastewater	USA	Ithaca	Largus Angenent
	4480764	WW_8	26450676	24728236	1516	0.006131					
	4480859	WW_9	20403845	19079713	937	0.004911					
	4480861	WW_10	24255570	22606160	1265	0.005596					
	4480863	WW_11	27922730	25983893	1349	0.005192					
	4480864	WW_12	27019804	25286960	1390	0.005497					
	4480865	WW_13	23457620	21845155	1156	0.005292					
	4480867	WW_14	31928594	29720208	1552	0.005222					
**Metagenomic data**	4494854	ww_15	11982170	11745881	941	0.008011	activated sludge	wastewater	China	Zhecheng Henan	Zhang Xu-xiang
	4494855	ww_16	11793394	11736958	989	0.008426					
	4494863	ww_17	11959298	11573478	835	0.007215					

	4494888	ww_18	11992160	11892576	1019	0.008568					
**Swiss WWTP metatranscriptomic**	4491800	ww_19	9455087	7251415	465	0.006413	activated sludge	wastewater	Switzerlan d		**David Johnson**
**Gut metagenome in European women with normal, impaired and diabetic glucose control**	ERR260267	H_gut_1	30242478	20471002	1114	0.005442	gut	human gut	Sweden		Intawat Nookaew
	ERR260266	H_gut_2	26467014	18857699	917	0.004863					
	ERR260265	H_gut_3	30765628	21343905	1765	0.008269					
	ERR260264	H_gut_4	33976795	23364674	1405	0.006013					
	ERR260263	H_gut_5	36334873	24513211	2014	0.008216					
	ERR260260	H_gut_6	26408258	18513044	1121	0.006055			France		
	ERR260259	H_gut_7	32535577	21985794	1439	0.006545			Germany		
	ERR260258	H_gut_8	20701781	13007871	2071	0.015921			Sweden		
	ERR260256	H_gut_9	24710958	14581521	1437	0.009855					
	ERR260255	H_gut_10	17052018	11059028	631	0.005706					
	ERR260253	H_gut_11	22105477	13414522	596	0.004443					
	ERR260252	H_gut_12	25148170	14844855	872	0.005874			Germany		
	ERR260251	H_gut_13	22708245	14133580	902	0.006382			Sweden		
	ERR260250	H_gut_14	28446475	16251634	1424	0.008762					
	ERR260244	H_gut_15	17354593	11316121	702	0.006204					
	ERR260243	H_gut_16	19796334	13120215	728	0.005549					
	ERR260242	H_gut_17	14410596	9143104	497	0.005436					
	ERR260234	H_gut_18	22455672	9673218	752	0.007774					
	ERR260231	H_gut_19	19028486	9555211	725	0.007587					
	ERR260230	H_gut_20	17352183	9154410	598	0.006532					
	ERR260227	H_gut_21	20661171	12519645	1088	0.00869					
	ERR260226	H_gut_22	25119730	15285719	641	0.004193					
	ERR260225	H_gut_23	17185502	10125862	491	0.004849					
	ERR260224	H_gut_24	25223709	16110123	953	0.005916					
	ERR260216	H_gut_25	7680054	4371482	369	0.008441					
	ERR260215	H_gut_26	12755195	7436083	372	0.005003					
	ERR260209	H_gut_27	22991191	14439795	650	0.004501					
	ERR260205	H_gut_28	25909440	14143268	887	0.006272					
	ERR260204	H_gut_29	23833078	14804488	827	0.005586			Iceland		

	ERR260193	H_gut_30	24617274	15028588	994	0.006614					
	ERR260180	H_gut_31	29040623	21010677	951	0.004526					
	ERR260175	H_gut_32	22116677	18388685	824	0.004481					
	ERR260171	H_gut_33	37363289	31122042	1884	0.006054			Sweden		
	ERR260170	H_gut_34	27832817	15371010	1586	0.010318					
	ERR260163	H_gut_35	27910666	16291081	804	0.004935					
	ERR260153	H_gut_36	25881584	13265822	841	0.00634					
	ERR260146	H_gut_37	19786858	13149682	1301	0.009894					
**Host remodeling of the gut microbiome and metabolic changes during pregnancy**	4474351	H_gut_38	23207774	20217593	1081	0.005347	feces	human gut	Finland	Turku	Ryth Ley
	4474352	H_gut_39	15766776	14105737	1172	0.008309					
	4474355	H_gut_40	24684997	21229509	1134	0.005342					
	4474357	H_gut_41	19531233	16840633	1404	0.008337					
	4474358	H_gut_42	13097192	11351497	2099	0.018491					
	4474359	H_gut_43	20385087	16526438	3696	0.022364					
	4474360	H_gut_44	24267385	21368764	1354	0.006336					
	4474361	H_gut_45	18900012	16979120	1723	0.010148					
	4474369	H_gut_46	24363633	20924499	1444	0.006901					
	4474805	H_gut_47	24449696	21071660	1523	0.007228					
**HMP**	SRS052697	H_gut_48	*Not indicated	43661492	7807	0.017881	feces	human gut	USA		
	SRS053214	H_gut_49		50800640	14798	0.02913					
	SRS056259	H_gut_50		53398256	17904	0.033529					
	SRS058770	H_gut_51		60825750	11750	0.019317					
	SRS013215	H_gut_52		50233735	6852	0.01364					
	SRS011239	H_gut_53		61916490	20336	0.032844					
	SRS013476	H_gut_54		44918049	3123	0.006953					
	SRS016495	H_gut_55		50003681	4830	0.009659					
	SRS022071	H_gut_56		48644274	12816	0.026346					
	SRS062427	H_gut_57		45215279	5927	0.013108					
	SRS024265	H_gut_58		24752602	4364	0.01763					
	SRS016989	H_gut_59		60289323	9621	0.015958					
	SRS019685	H_gut_60		49806486	11583	0.023256					

	SRS050422	H_gut_61		36569518	4590	0.012551					
	SRS058723	H_gut_62		55828003	15689	0.028102					
	SRS063985	H_gut_63		47633751	9624	0.020204					
**Bovine metagenome**	4519873	A_gut_1	5501350	5463738	75	0.001373	bodily fluid	bovine gut	Australia	Victoria	Elizabeth Ross
	4519885	A_gut_2	8803493	8729548	1251	0.014331	feces				
	4519888	A_gut_3	5378316	5345758	62	0.00116	bodily fluid				
	4519890	A_gut_4	6321628	6203557	410	0.006609	feces				
	4519892	A_gut_5	4842154	4810905	446	0.009271	feces				
	4519894	A_gut_6	5712271	5658658	58	0.001025	bodily fluid				
	4519896	A_gut_7	5381779	5349573	509	0.009515	feces				
	4519897	A_gut_8	4524511	4489934	59	0.001314	bodily fluid				
	4520059	A_gut_9	5425084	5381608	99	0.00184	bodily fluid				
	4520065	A_gut_10	5498955	5469586	83	0.001517	bodily fluid				
	4520069	A_gut_11	4910725	4867006	61	0.001253	bodily fluid				
	4520073	A_gut_12	5719309	5668775	55	0.00097	bodily fluid				
	4520077	A_gut_13	5448846	5414071	32	0.000591	bodily fluid				
	4520079	A_gut_14	4521509	4494362	52	0.001157	bodily fluid				
	4520083	A_gut_15	3658097	3630470	66	0.001818	bodily fluid				
	4520087	A_gut_16	5601728	5567072	66	0.001186	bodily fluid				
	4520089	A_gut_17	3829112	3797221	62	0.001633	bodily fluid				
	4520091	A_gut_18	3582414	3558251	33	0.000927	bodily fluid				
	4520093	A_gut_19	4076094	4032094	41	0.001017	bodily fluid				
	4520095	A_gut_20	3589425	3567550	44	0.001233	bodily fluid				
	4520061	A_gut_21	7175457	7107860	657	0.009243	feces				
	4520063	A_gut_22	3815729	3794254	327	0.008618	feces				
	4520067	A_gut_23	5161808	5121473	609	0.011891	feces				
	4520071	A_gut_24	5295240	5263252	734	0.013946	feces				
	4520075	A_gut_25	4778871	4733779	405	0.008556	feces				
	4520081	A_gut_26	4427042	4392558	365	0.00831	feces				
	4520085	A_gut_27	4726808	4667764	578	0.012383	feces				

Table S2. Selected metagenomes used in phylogenetic analysis according environment.

**Environment**	**Metagenomes randomly selected (for more information see Sup table 1)**	**Number of metagenomes**
Ocean	ocean_4, ocean_8, ocean_11, ocean_15, ocean_19, ocean_20, ocean_21	7
Fresh water	fresh_w_1, fresh_w_3, fresh_w_6	3
Non-agricultural soils	und_3, und_5, und_8, und_11, und_17, und_23, und_31, und_35, und_37, und_49, und_55,	11
Agricultural soils	dis_4, dis_7, dis_11, dis_15, dis_19, dis_21, dis_24,	7
Bovine rumen	a_gut_3, a_gut_6, a_gut_11,	3
Bovine feces	a_gut_2, a_gut_6, a_gut_22, a_gut_25	4
Human gut	h_gut_3, h_gut_4, h_gut_13, h_gut_14, h_gut_19, h_gut_23, h_gut_25, h_gut_33, h_gut_38, h_gut_39, h_gut_43, h_gut_47,	12
Wastewater (aerobic and anaerobic)	ww_1, ww_2, ww_4, ww_17, ww_19,	5

Table S3. β-lactamase gene network statistics.

**Network Analysis**	
Network type	undirected
cc Double (clustering coefficient)	0.73496617
ncc Integer (connected components)	5
diameter Integer (network diameter)	12
radius Integer (network radious)	1
centralization Double (network centralization)	0.198043881
connPairs Long (Shortest path)	34062
avSpl Double (Characteristic path length)	4.537784041
avNeighbors Double (Average number of neighbors)	27.7755102
nodeCount Integer (number of nodes)	196
density Double (network density)	0.142438514
heterogeneity Double (network heterogeneity)	0.736863613

Table S4. Clusters detected in network analysis.

**Clustering Results: 8 complexes in all**	
**Parameters:**<br>**Network Scoring:** Include Loops: true, Degree Cutoff: 2<br>**Cluster Finding:** Node Score Cutoff: 0.2, Haircut: true, Fluff: true, Fluff Density, Cutoff 0.1<br>K-Core: 2, Max. Depth from Seed: 100	
**Complex 1**	dis11, dis22, dis19, ww15, dis7, und19, und26, dis23, glac3, und25, dis6, und20, ww3, ww6, und10, dis24, glac5, ww4, glac2, dis5, fresh7, und49, dis12, ocean20, ww5, und2, dis9, glac10, dis4, und15, und8, und56, und33, und41, dis20, fresh11, und36, und42, glac4, glac1, und29, und35, dis3, und43, und18, und54, ocean22, ww18, und39, und52, und34, dis8, und48, dis10, dis2, und47, und37, dis14, und38, ww19, und44, und40, und46, ww16, ww17, ocean21, und7, und1, und12, und21, und4, und16, und13, fresh9, und30, und17, und5, dis21, und24, und31, ww2, und14, und11, und50, und9, und23, und51, dis13, und45, dis18, glac7, und28, ocean19, fresh10, und6, ocean18, und53, glac8, glac9, und55, und3, glac6, und22, und27, fresh8
**Complex 2**	ww14, ww12, ww9, ww11, ww10, ww13, ww7, ww8
**Complex 3**	Agut24, Agut5, Agut23, Agut22, Agut21, Hgut25, Agut26, Agut7, Agut27, Agut25, Agut4
**Complex 4**	Hgut13, Hgut45, Hgut35, Hgut9, Hgut26, Hgut6, Hgut14, Hgut32, Hgut33, Hgut23, Hgut17, Hgut7, Hgut3, Hgut10, Hgut46, Hgut1, Hgut22, Hgut24, Hgut36, Hgut15, Hgut29, Hgut11, Hgut4, Hgut21, Hgut38, Hgut54, Hgut18, Hgut20, Hgut16, Hgut37, Hgut41, Hgut40, Hgut12, Hgut19, Hgut5, Hgut47,<br>Hgut39, Hgut30, Hgut28, Hgut34, Hgut44, Hgut2, Hgut31, Hgut27
**Complex 5**	Hgut63, Hgut60, Hgut58, Hgut57, Hgut51, Hgut43, Hgut55, Hgut61, Hgut53, Hgut56, Hgut50, Hgut59, Hgut48, Hgut49, Hgut62
**Complex 6**	Agut17, Agut13, Agut1, Agut8
**Complex 7**	Agut12, Agut11, Agut15
**Complex 8**	fresh1, fresh2

Table S5. Cluster I statistics.

Name	Average Shortest Path Length	Betweenness Centrality	Closeness Centrality	Clustering Coefficient	Degree	Eccentricity	MCODE Score	Neighborhood Connectivity	Number Of Directed Edges	Radiality	Stress	Topological Coefficient
und44	2.769231	0.003212	0.361111	0.8	10	4	8	16.2	10	0.705128	2518	0.476471
und48	2.403846	0.001243	0.416	0.808333	16	4	11	22.8125	16	0.766026	980	0.47526
und33	2.509615	0.002599	0.398467	0.747253	14	4	8.590909	18.5	14	0.748397	3338	0.420455
und41	2.326923	0.077542	0.429752	0.561905	21	4	11	18.95238	21	0.778846	76016	0.371615
und42	2.038462	0.016455	0.490566	0.626667	25	3	10.04211	26.32	25	0.826923	13152	0.350933
und35	2	0.016049	0.5	0.671937	23	3	10.24561	27.43478	23	0.833333	14146	0.338701
und43	2.557692	0.01277	0.390977	0.747253	14	4	9.69697	18.21429	14	0.740385	11700	0.433673
und39	2.278846	0.033524	0.438819	0.610526	20	4	10.51648	20.9	20	0.786859	25462	0.379091
und40	2.586538	0.006168	0.386617	0.74359	13	4	8.836364	18	13	0.735577	6340	0.45
und45	3.221154	0.047892	0.310448	0.357143	8	5	4	8	8	0.629808	41780	0.333333
und36	1.913462	0.026588	0.522613	0.616092	30	3	16	31.53333	30	0.847756	28688	0.37992
und34	2.201923	0.01715	0.454148	0.628571	21	4	9.9	23.28571	21	0.799679	12172	0.369615
und38	1.884615	0.028463	0.530612	0.649573	27	3	13.35948	33.81481	27	0.852564	30808	0.379942
und46	3.115385	0.001081	0.320988	0.75	8	5	6	13.125	8	0.647436	214	0.504808
und47	3.25	0.009072	0.307692	0.6	5	5	2.7	10.4	5	0.625	13278	0.433333
ocean22	3.346154	0	0.298851	1	4	5	4	6.5	4	0.608974	0	0.65
ocean18	3.346154	0	0.298851	1	4	5	4	6.5	4	0.608974	0	0.65
ocean19	2.442308	0.01648	0.409449	0.535714	8	4	2.555556	15.875	8	0.759615	6586	0.293981
ocean21	2.778846	0.002562	0.359862	0.8	5	5	4	11.4	5	0.703526	1796	0.335294
ocean20	2.5	0.019967	0.4	0.527778	9	5	2.488889	19.22222	9	0.75	11264	0.369658
und28	1.548077	0.026166	0.645963	0.653005	61	4	35	49.7377	61	0.908654	27414	0.534814
ww16	1.913462	0.010714	0.522613	0.673846	26	4	15.89542	38.38462	26	0.847756	6894	0.426496
glac8	1.788462	0.003969	0.55914	0.771772	37	4	23.85231	48.05405	37	0.86859	3292	0.522327

ww18	1.701923	0.034696	0.587571	0.606272	42	4	22.57538	40.7381	42	0.883013	20524	0.424355
und18	1.673077	0.064756	0.597701	0.62237	43	4	22.33069	43.86047	43	0.887821	41354	0.447556
und27	1.538462	0.024211	0.65	0.69064	62	4	37	51.37097	62	0.910256	22936	0.552376
dis11	1.663462	0.019184	0.601156	0.684397	48	4	27.54839	48.83333	48	0.889423	20044	0.519504
ww19	2.230769	0.008465	0.448276	0.730994	19	4	11.73626	26.31579	19	0.794872	13018	0.424448
dis10	1.75	0.031928	0.571429	0.6875	33	3	19	41	33	0.875	32144	0.42268
dis20	1.730769	0.036207	0.577778	0.663866	35	3	18.24275	40.77143	35	0.878205	35148	0.420324
glac3	1.673077	0.014151	0.597701	0.680851	48	4	27.60484	48.16667	48	0.887821	12100	0.517921
und25	1.644231	0.014893	0.608187	0.729412	51	4	31.64444	52.31373	51	0.892628	20122	0.562513
ww3	1.586538	0.016857	0.630303	0.682642	59	4	35	50.47458	59	0.902244	13110	0.554666
und10	1.538462	0.024573	0.65	0.707837	64	4	37.95128	51.96875	64	0.910256	20298	0.571085
glac2	1.663462	0.015331	0.601156	0.662415	49	4	27.60484	47.44898	49	0.889423	12368	0.510204
fresh7	1.798077	0.007689	0.55615	0.730612	50	5	32	51.52	50	0.866987	3600	0.606118
glac4	1.759615	0.013283	0.568306	0.668016	39	4	21.70667	41.74359	39	0.873397	8344	0.448856
und2	1.576923	0.029384	0.634146	0.68797	57	4	34	51.05263	57	0.903846	31046	0.543113
fresh9	2.153846	3.39E-04	0.464286	0.813187	14	5	11	54.28571	14	0.807692	164	0.646259
und37	1.788462	0.033785	0.55914	0.643494	34	3	17.37662	37.91176	34	0.86859	33588	0.412084
ww17	2.423077	9.08E-04	0.412698	0.75	9	4	7	24.44444	9	0.762821	208	0.444444
ww2	3.269231	0	0.305882	0	1	5	0	20	1	0.621795	0	0
ww15	1.846154	0.037478	0.541667	0.612903	32	4	17.81053	36.90625	32	0.858974	18254	0.405563
dis19	1.846154	0.015587	0.541667	0.726154	26	3	15.41053	39.34615	26	0.858974	17038	0.418576
und20	1.528846	0.024898	0.654088	0.685781	66	4	38.90488	51.01515	66	0.911859	18970	0.566835
glac5	2.221154	0.001479	0.450216	0.863636	12	3	8.454545	33.66667	12	0.796474	1516	0.487923
dis12	1.634615	0.020069	0.611765	0.698039	51	4	29.97598	50.54902	51	0.894231	22454	0.537756
dis21	2.201923	9.06E-05	0.454148	0.984146	41	6	37.86098	53.04878	41	0.799679	216	0.791773
und53	1.932692	6.67E-04	0.517413	0.939626	49	5	39.73422	54.61224	49	0.844551	2666	0.718582
und55	1.990385	3.20E-04	0.502415	0.965217	46	5	39.73422	54.28261	46	0.834936	1348	0.723768
und3	1.894231	0.00102	0.527919	0.92	51	5	39.73422	54.78431	51	0.850962	3760	0.702363
und22	1.836538	0.001889	0.544503	0.878788	55	5	39.73422	54.49091	55	0.860577	5798	0.689758
fresh8	1.990385	3.20E-04	0.502415	0.965217	46	5	39.73422	54.28261	46	0.834936	1348	0.723768

und52	2.028846	2.46E-04	0.492891	0.970707	45	5	39.73422	54.08889	45	0.828526	998	0.740944
und50	2.461538	0	0.40625	1	31	6	31	51.64516	31	0.75641	0	0.846642
und7	1.817308	0.00227	0.550265	0.867532	56	5	39.73422	54.41071	56	0.863782	6764	0.680134
und1	1.788462	0.003293	0.55914	0.838476	58	5	39.73422	54.08621	58	0.86859	8268	0.667731
und12	1.759615	0.004669	0.568306	0.808197	61	5	39.73422	53.54098	61	0.873397	9860	0.661
und19	1.615385	0.013517	0.619048	0.741827	65	5	39.73422	52.07692	65	0.897436	10454	0.598585
und21	1.769231	0.004026	0.565217	0.822034	60	5	39.73422	53.88333	60	0.871795	9636	0.665226
und4	1.778846	0.003112	0.562162	0.832846	59	5	39.73422	54.28814	59	0.870192	4506	0.670224
und16	1.778846	0.003455	0.562162	0.832846	59	5	39.73422	54	59	0.870192	8402	0.666667
und13	1.653846	0.007443	0.604651	0.791116	62	5	39.73422	53.45161	62	0.891026	7944	0.62153
und51	2.096154	1.14E-04	0.477064	0.982578	42	5	38.81882	53.33333	42	0.817308	392	0.761905
und49	1.942308	5.51E-04	0.514851	0.949468	48	5	39.73422	54.5625	48	0.842949	2320	0.717928
und30	1.798077	0.003059	0.55615	0.844525	58	5	39.73422	54.10345	58	0.866987	8142	0.676293
und17	1.798077	0.003059	0.55615	0.844525	58	5	39.73422	54.10345	58	0.866987	8142	0.676293
und8	1.865385	0.001322	0.536082	0.909502	52	5	39.73422	54.80769	52	0.855769	4728	0.693768
und56	1.942308	5.51E-04	0.514851	0.949468	48	5	39.73422	54.5625	48	0.842949	2320	0.717928
und5	1.759615	0.004133	0.568306	0.804372	61	5	39.73422	53.63934	61	0.873397	5006	0.662214
und24	1.778846	0.003417	0.562162	0.822911	59	5	39.73422	54.01695	59	0.870192	4546	0.666876
und31	1.701923	0.005942	0.587571	0.83584	57	5	39.73422	54.45614	57	0.883013	7192	0.633211
und14	1.644231	0.008248	0.608187	0.777266	63	5	39.73422	53.04762	63	0.892628	8150	0.616833
und11	1.644231	0.008248	0.608187	0.777266	63	5	39.73422	53.04762	63	0.892628	8150	0.616833
und29	1.932692	6.67E-04	0.517413	0.939626	49	5	39.73422	54.61224	49	0.844551	2666	0.718582
und9	1.817308	0.00227	0.550265	0.867532	56	5	39.73422	54.41071	56	0.863782	6764	0.680134
und54	2	3.04E-04	0.5	0.966667	45	5	38.81882	54.44444	45	0.833333	1340	0.725926
und23	1.769231	0.004026	0.565217	0.822034	60	5	39.73422	53.88333	60	0.871795	9636	0.665226
und26	1.538462	0.024758	0.65	0.728671	66	4	38.3697	52.22727	66	0.910256	16520	0.586823
fresh10	1.932692	0.001381	0.517413	0.914007	48	5	37.39746	54.6875	48	0.844551	1018	0.710227
dis22	2.076923	1.39E-04	0.481481	0.980066	43	5	39.73422	53.60465	43	0.820513	468	0.754995
dis24	2.057692	1.85E-04	0.485981	0.975687	44	5	39.73422	53.84091	44	0.823718	706	0.74779
fresh11	1.942308	0.001275	0.514851	0.925069	47	5	38.3697	54.25532	47	0.842949	718	0.704615

und15	1.548077	0.021318	0.645963	0.711058	65	4	38.81882	51.78462	65	0.908654	17026	0.58185
und6	1.605769	0.013742	0.622754	0.715668	58	4	34.80085	51.82759	58	0.899038	14226	0.575862
glac7	1.846154	0.002099	0.541667	0.828877	34	4	23.92615	50.85294	34	0.858974	1266	0.571381
dis7	1.538462	0.024327	0.65	0.700687	62	4	36.95007	51.70968	62	0.910256	22206	0.556018
glac9	1.692308	0.005352	0.590909	0.783163	49	4	32.79048	53.28571	49	0.884615	5196	0.592063
glac6	1.807692	0.004404	0.553191	0.788889	45	5	28.87387	52.64444	45	0.865385	2624	0.605109
dis9	1.836538	0.001861	0.544503	0.876768	55	5	39.73422	54.38182	55	0.860577	5198	0.688377
dis8	1.884615	0.001183	0.530612	0.908748	52	5	39.73422	54.67308	52	0.852564	3894	0.700937
glac10	1.894231	9.91E-04	0.527919	0.876847	29	4	22.57538	49.93103	29	0.850962	632	0.561023
dis23	2.096154	1.11E-04	0.477064	0.984146	41	5	37.86098	53.95122	41	0.817308	454	0.759876
dis6	1.836538	0.001861	0.544503	0.876768	55	5	39.73422	54.38182	55	0.860577	5198	0.688377
ww4	2.163462	2.96E-04	0.462222	0.901961	18	5	13.45098	47	18	0.80609	122	0.580247
ww6	2.317308	2.61E-05	0.431535	0.977778	10	5	8.836364	40	10	0.780449	12	0.56338
dis5	1.826923	0.002107	0.547368	0.867532	56	5	39.73422	54.30357	56	0.862179	5942	0.687387
dis4	1.807692	0.002489	0.553191	0.857143	57	5	39.73422	54.22807	57	0.865385	6908	0.677851
dis3	1.836538	0.001889	0.544503	0.878788	55	5	39.73422	54.49091	55	0.860577	5798	0.689758
dis2	1.605769	0.015598	0.622754	0.711888	66	5	38.69767	51.28788	66	0.899038	8450	0.589516
ww5	2.153846	1.63E-04	0.464286	0.933333	16	5	13.76667	43.375	16	0.807692	52	0.528963
dis14	4.192308	0	0.238532	1	2	6	0	6.5	2	0.467949	0	0.722222
dis13	4.201923	0	0.237986	1	2	6	0	5	2	0.466346	0	0.625
dis18	4.201923	0	0.237986	1	2	6	0	5	2	0.466346	0	0.625
glac1	3.038462	0	0.329114	1	5	5	5	19	5	0.660256	0	0.655172

Table S6. Nodes related to cluster I, with the high connectivity parameters.

name	Average Shortest Path Length	Betweenness Centrality	Closeness Centrality	Clustering Coefficient	Degree	Eccentricity	MCODE Score	Neighborhood Connectivity	Number Of Directed Edges	Radiality	Stress	Topological Coefficient
und28	1.548077	0.026166	0.645963	0.653005	61	4	35	49.7377	61	0.908654	27414	0.534814
und27	1.538462	0.024211	0.65	0.69064	62	4	37	51.37097	62	0.910256	22936	0.552376
und25	1.644231	0.014893	0.608187	0.729412	51	4	31.64444	52.31373	51	0.892628	20122	0.562513
glac2	1.663462	0.015331	0.601156	0.662415	49	4	27.60484	47.44898	49	0.889423	12368	0.510204
und55	1.990385	3.20E-04	0.502415	0.965217	46	5	39.73422	54.28261	46	0.834936	1348	0.723768
und22	1.836538	0.001889	0.544503	0.878788	55	5	39.73422	54.49091	55	0.860577	5798	0.689758
fresh8	1.990385	3.20E-04	0.502415	0.965217	46	5	39.73422	54.28261	46	0.834936	1348	0.723768
und1	1.788462	0.003293	0.55914	0.838476	58	5	39.73422	54.08621	58	0.86859	8268	0.667731
und4	1.778846	0.003112	0.562162	0.832846	59	5	39.73422	54.28814	59	0.870192	4506	0.670224
und30	1.798077	0.003059	0.55615	0.844525	58	5	39.73422	54.10345	58	0.866987	8142	0.676293
und56	1.942308	5.51E-04	0.514851	0.949468	48	5	39.73422	54.5625	48	0.842949	2320	0.717928
und31	1.701923	0.005942	0.587571	0.83584	57	5	39.73422	54.45614	57	0.883013	7192	0.633211

Table S7. Blastx against non-redundant NCBI database and comparison with blastx against EX-B database.

Project	read	Sequence	EX-B database		NCBI		Similarity to b-lactamase or antibiotic resistance homologue		
			Hit in our database	% of similarity	Best hit in NCBI	% of similarity	Best b-lactamase/antibiotic resistance related hit	% of similarity	Position of best b-lactamase hit
wwtp hkulg	>FCD1JGEACXX:4:1107:20509:10014#TACTATGA	CTGCGTTGGATCGCGGTGGCCATGTCCATGGCCGTGTCCCATTCGGGTCTGCGTTCGGTACCATCCCATGCGATCACCGTGTGCTCGTCCTCCACGGACCCGGTCTCCAGTGCTACGAGCGAATTGAATACCTTGAAAGTGGAAGCAGGCAGGAAACCCCTGTCG	blaOXA 62	51.02	class D beta-lactamase [Pseudanabaena sp. SR411]	62	class D beta-lactamase	62	1
	>FCD1JGEACXX:4:1104:17488:11921#TACTATGA	CTGATCTTCTCGCGGTTCTTGCTTTGAGAACCGGTCCCAAACAGCCTTGGCGACCTTCGCGATGACCCTTTCGCGGGTCTTCTCGTCGGCGGCCGAGTCACCGACAAAGACCGCTATCAACAGGTGCCGTCCGCCCGGCAGCGTAATGATGCCGATGTCG	blaPER 2	58.54	ClassA_beta_lactamase [uncultured bacterium]	76	ClassA_beta_lactamase [uncultured bacterium]	76	1
	>FCD1JGEACXX:4:1106:12171:5297#TACTATGA	AAATTCAAAACTTACCATAGCTCACCTAACCGGCAACTTTTATATTTACACTACCTATAATACTTATGAGGAAAATAAAATTCCCGCAACCGGGATGTATCTTGTAGCTAAGGATGCTGTGGTATTATTTGACACACCCTGGGATACTACACAGTTTCAACCCC	blaJOHN 1	61.11	BlaB/IND/MUS family subclass B1 metallo-beta-lactamase [Flavobacterium sinopsychrotolerans]	79	BlaB/IND/MUS family subclass B1 metallo-beta-lactamase [Flavobacterium sinopsychrotolerans]	79	1
	>FCD1JGEACXX:4:1204:10623:61440#TACTATGA	CGTGAAGCGGGACATCGAGTCGTGGAACGGCGACCAGGACCTGCGCTCCTCGATGCGCAACTCGGTCGTCTGGGTCTACCAGCGGATCGCGCGGGACATCGGCGAGGAACGCGAGCGTCGCTACCTGGAGAACATCGACTACGGGAATGCCGAGCCCTCCGGCGGAATCGACCGGTTC	blaOXA 119	64.15	ClassD_beta_lactamase [uncultured bacterium]	73	ClassD_beta_lactamase [uncultured bacterium]	73	1
	>FCD1JGEACXX:4:1116:3621:46690#TCCTATGA	CCGCGCGCTGCGCGCGCCGTCCTGCGGAAGGATTCATCGGTCGATCGCATACTTTCTCACCACTGTGTTGATGTCCGGCGACTGGTCTTAGGGCATGGCATCGATGGAGCGCAGGATCGCACGGGCAATTCCCTCGCGCTTCGGCAGGTCCGCGCTGCCGTTCGGCA	blaOXA 46	76	ClassD_beta_lactamase [uncultured bacterium]	72	ClassD_beta_lactamase [uncultured bacterium]	72	1
Soil microbiome analysis along a precipitation gradient	>MG00HS16:732:HYLC2ADXX:2:1110:3929:93333	CGCCGCCCGCGCGATCTCCGCGATCAACCGCTCCTTCGCGGCCTCGCCCGTCTTCAGATCTTCGGTGAACACGGCAATGGCCAGCCGGCCGCCTGACGGAAGGTCGACCAGGCCGACGTCGTTGACCACGACCGTCCCATAAGTTCCCGTCTTGACGGCGACGACGGTGCCCTTCGACAACAGCGCCCGGATCCGGTTGGTTCCGGTCTCGTCCCGGCGGAGGATCT	blaLRA 5	56.58	serine hydrolase [Eudoraea adriatica]	55	class A beta-lactamase [Cytophagaceae bacterium BCCC1]	48	4
A human gut catalog	>ERR011110.4056072_I328_1_FC30MD2AAXX:5:36:1410:165_1	TGCGCTATACTCTTTCCCAGAGCGACAATAATGCAAGCAATATCATGTTTAAGAATATGCTCAATACTGCACAAACAGACAGTTTTATAGCGAAACTCATACCACATTCGAGTTTTCAGATAGCTTA	CfxA 4	82.93	beta-lactamase [Prevotella copri CAG:164]	100	beta-lactamase [Prevotella copri CAG:164]	100	1

	>ERR011110.10208834_I328_1_FC30MD2AAXX:5:91:1147:1965_1	GCGACCGTAATGCGAAAGGACAACAGATCGGTTGCAATGACATCGGGTTTATTCTTCTTCCCGACGGACATGCCTATAGTATAGCCGTCTTCGTGAAAGATTCCGAAGCAGATAACAGAGAAAACAGTGA	Cephalosporinase	100	epA family class A extended-spectrum beta-lactamase [Bacteroides fragilis]	92	epA family class A extended-spectrum beta-lactamase [Bacteroides fragilis]	92	1
	>ERR011198.8566863_I354_1_FC30M2LAAXX:7:74:1407:1632_1	GCCGTAGTGCAGCTTTTTAATGTTGGCCTGCATGCGTTCTGTGCCGGTGCGGCGCGCAAGTTCTTTATAGGCGGGCACTTGCGAGAGTTTCATGGCGCTGCGCAGGCTGGCGTC	blaOXA 192	65.79	class D beta-lactamase [Phascolarctobacterium sp. CAG:266]	95	class D beta-lactamase [Phascolarctobacterium sp. CAG:266]	95	1
	>ERR011198.1629008_I354_1_FC30M2LAAXX:7:14:491:1826_1	GTTTGTTTACAGAAAGTCTTATCAGTCAAGAGAAACAAGATTTCATTAAGAATGCATTGAAAGAATGTAAAACAGGTATAGATAGGATAGTAGCTCCACTTCTTGA	CfxA 4	79.41	beta-lactamase [Prevotella copri CAG:164]	97	beta-lactamase [Prevotella copri CAG:164]	97	1
	>ERR011198.11494165_I354_1_FC30M2LAAXX:7:100:800:186_1	CTTGATATTGGAAAAGAATATCACAAGCATTATTGTCACTCTGTTGAAGTGTATATCTTAATAGATCGGCAATGCTTATTTCAAAATCTCCTCGTGGATACTTTTCTCGTAAGGGGCTATAGGTATC	Beta-lactamase	83.33	Beta-lactamase enzyme family protein [Bacteroides xylanisolvens]	83	Beta-lactamase enzyme family protein [Bacteroides xylanisolvens]	83	1
	>ERR011198.5355365_I354_1_FC30M2LAAXX:7:45:528:283_1	AAAGTTCGAAGGCACTCCAAGGCAATTTTTTCACGCAGCGGCAAATCTTTGGCGTACGGCATCCAAGTTCAGCGCGAAAACGTATAGGTTATCTTTGGCTTCCACCCAGCCCACAAACCAGCCTAC	blaOXA 85	57.89	OXA-63 family oxacillin-hydrolyzing class D beta-lactamase OXA-478 [Brachyspira pilosicoli]	67	OXA-63 family oxacillin-hydrolyzing class D beta-lactamase OXA-478 [Brachyspira pilosicoli]	67	1
	>ERR011198.6917817_I354_1_FC30M2LAAXX:7:59:640:939_1	TAGGATATGGCAATAAAAATATATCCGGAGATTTCTCTTCCTACTGGATGCAGTCATCCT TAAAGATTTCACCAGTAGAACAAGTTGAACTTTTGAAAAATTTATATCGAAACAACTTCAACTTTAC	mecR1	57.5	hypothetical protein [[Ruminococcus] torques]	100	BlaR1 family beta-lactam sensor/signal transducer [Lachnotalea glycerini]	78	8
www metagenomic data	>FCD0RBVACXX:8:1201:20087:97365#GCGGAACT/1	GGCGTCGCGGACCTGACGCGAGGCACCCCGATCACCCCGGATACCGGCGTTCGGATCGCCTCGATCAGCAAGACGTTCACCGCGGTCGCGGCGATGCAGG	Beta-lactamase	56.67	hydrolase [Nocardia carnea]	67	CubicO group peptidase, beta-lactamase class C family [Arachidicoccus rhizosphaerae]	55	23
	>FCD0RBVACXX:8:1201:5778:83336#GCGGAACT/2	TATTGTTGATCTATCTTTCAAAAGTTTATCGGGAATGACGGGCGTAAAGGTTTCGCCGTATTTTCCCAACGCATAATCGGTTTTTCCGCCGGATTTCAAA	blaGOB 16	63.64	GOB family subclass B3 metallo-beta-lactamase [Pedobacter agri]	70	GOB family subclass B3 metallo-beta-lactamase [Pedobacter agri]	70	1

	>FCD0RBVACXX:8:1202:13164:18326#GCGGAACT/2	CAACGTACCAGCCCACCGAGGGGGTGCTGCCGGTCGCCCATCCCGTCTTTGCATAAAGGCGATACCGAGCAGTCTCATCGCTGAGCATGATTTTCTTCAG	LCR 1	78.79	classD [uncultured bacterium]	82	classD [uncultured bacterium]	82	1
	>FCD0RBVACXX:8:1202:19825:21099#GCGGAACT/2	ACAAGGTCAGCGACTACATCGCGGACATGAAGGGCTCGGCCTACGACGACGTGAGCATCCGCCAGTTGCTGACGATGACCTCGGGCATCCGGTGGAACGA	Cephalosporinase	53.57	serine hydrolase [Lautropia sp. SCN 70-15]	100	CubicO group peptidase, beta-lactamase class C family [Variovorax sp. OV329]	84	8
	>FCD0RBVACXX:8:1202:3032:61052#GCGGAACT/2	ATTCTCGATCAGATCGCCGGTGAACAGCACATCGGCATCGGGAACGTACGCAACCAGGTCGGCCGGTGAGTGGGCATGCCCCAAATGCAGAAGTTCAATC	blaVIM 33	57.14	MBL fold metallo-hydrolase [Nitratireductor basaltis]	63	MBL fold metallo-hydrolase [Nitratireductor basaltis]	63	1
	>FCD0RBVACXX:8:1203:12561:93885#GCGGAACT/2	ATGCTCACCGATGAGACTGCTCGGTATCGCATTTATGCAAAGACGGGATGGGCGACCGGCAGCACCCCCTCGGTGGGCTGGTACGTTGGCTATGTTGAGG	LCR 1	78.79	classD [uncultured bacterium]	79	classD [uncultured bacterium]	79	1
	>FCD0RBVACXX:8:1205:8235:133659#GCGGAACT/2	CGATGCCGCCGGTGGCAAATGTCGAAGTGGTGCGGGACCGGGAAGTCGTGAAGCTGGGTTCGCTCGAACTGACCATGCACGCTACACCCGGGCATACGCC	Beta-lactamase	65.62	metallo beta-lactamases [Massilia sp. SB1-3]	75	metallo beta-lactamases [Massilia sp. SB1-3]	75	1
	>FCD0RBVACXX:8:1207:15919:55744#GCGGAACT/2	ATGTCCCCGAACAGACCGGTATCCTGCGCAAAATAGATCTTCTTCGCGTCGTTGGTGGTTAGCAGGAAGCCCGCCGGGTTGCCGCCGTTCGAGCCGTCGG	Metallo-b-lactamase	70.83	metal-dependent hydrolase [Chloroflexi bacterium HGW-Chloroflexi-6]	91	metal-dependent hydrolase [Chloroflexi bacterium HGW-Chloroflexi-6]	91	1
	>FCD0RBVACXX:8:1207:6573:102201#GCGGAACT/2	CGTTTGCGCGAAGGAAGCGCGTGAAACCGTCCGGCCCGCCTTGAATCTCCAAAAGCAGATTGGCGGCGGTGTTGTCGCTCATAACGACAGCGCCGGCGCA	blaOKPB 8	70.97	class A beta-lactamase [Paracoccus saliphilus]	70	class A beta-lactamase [Paracoccus saliphilus]	70	1
	>FCD0RBVACXX:8:1208:16529:7467#GCGGAACT/2	GTCCGGTAGCCAGACCGAGATGTCGCCCGGGCTGTGTGCCGGGCCGAGGTAGAGCAGCTCGATCCGTTTGCCGCCCATCTCGATGACCTTGCGCTCCTCG	blaIMP 7	52.17	sulfurtransferase [Thiotrichaceae bacterium]	78	MBL fold metallo-hydrolase [Dinoroseobacter shibae]	79	3
	>FCD0RBVACXX:8:1301:18017:63863#GCGGAACT/2	ATTCGCAATCGAATACCGGGTACCGTTCCAGTGAAAGGTCGAGTCGTCGCCTGCAATGGCACCCTCTTCAAGAGCAATCAAAGTATTGAGCACCTTGAAC	LCR 1	81.82	oxacillin-hydrolyzing class D beta-lactamase LCR-1 [Pseudomonas aeruginosa]	82	oxacillin-hydrolyzing class D beta-lactamase LCR-1 [Pseudomonas aeruginosa]	82	1
	>HWI-ST1047:65:D0NJ9A	CTTCTTTATCAAGAAGTGGAGCTGCTATCCTATCTACACCTGTTTTGCATTCTTTTAACGTATTCTTAATGAAACTTTGTTTCTCATCATCGATA	CfxA 4	100	putative CfxA protein	100	putative CfxA protein	100	1

Bovine metagenome	CXX:5:1206:3545:68192				[Parabacteroides distasonis]		[Parabacteroides distasonis]		
	>HWI-ST1047:65:D0NJ9ACXX:5:2208:7775:168550	CATCATTGTGAGCTGCAAGAACACCATTTTCATTAACATAACCTGAACCTGTCTTATGCGCTATAACAACCCCTTCTTTATCAAGAAGTGGAGCT	CfxA 2 like	100	putative CfxA protein [Bacteroides vulgatus]	100	putative CfxA protein [Bacteroides vulgatus]	100	1
Rootmosrner glacier cryogonite metagenome	>9226509 92 152	ATCCGGCGCGCGATCTCCTGATACACCGGCACCGCCGAGGCGGCGATCGCCGAGCGCAGCGTGTGATCCTGATTCCACGCCGCGATCGCGCG	blaOXA 356	66.67	class D beta-lactamase [Bradyrhizobium sp. URHA0002]	90	class D beta-lactamase [Bradyrhizobium sp. URHA0002]	90	1
	>8942535 100 214	GTGTTTAGGATTCACGCCGTAGATCGGCCCCGCAAAATGACCGGCACGCAGGTTGCGCCAGACCGTCGCGCCCACGCTGGCGGCCCGGTCCGATGCCCCA	blaSHV 112	55.56	acetyltransferase [Variovorax sp. CF079]	85	acetyltransferase [Variovorax sp. CF079]	85	1
	>11805719 167 320	TGGACGCTGTCCGGAATCTGCAACGGAAAATCGCCTGCGGTATGCGTGCCCAGATTCAGCAGGCTGATTTTGTCAAAGCTGGTGCCACGCAAATCCGGCAAATACGTGCTGACGCTATCCGACAGTTTTAGCTGTCCGTTAACCTCAGCATAGCTGGCCAGGGTGGC	blaLRA 13	72.73	class C beta-lactamase [Candidatus Regiella insecticola]	82	class C beta-lactamase [Candidatus Regiella insecticola]	82	1
ocean sediments	>FCC07FEACXX:8:1102:16123:16971#ACTTGAAT/1	CCTCGAAGCTCTCGTGACTCACCAGACGCCCCTCGTCCACGCGCGTCGAGTCCAACAGCACCTGAATCCAGCGGGCCATGTCCGACACACTGGAGTACAT	blaCMY 73	62.50	beta-lactamase (plasmid) [Gemmatirosa kalamazoonesis]	59	beta-lactamase (plasmid) [Gemmatirosa kalamazoonesis]	59	1
	>FCC07FEACXX:8:1104:19430:162869#ACTTGAAT/1	ATCCGGACGCTCTCCGACGAGCCCATCCGTTTTCTCATCAATACCCACATGCATCCGGACCACACGGGCGGAAACGAGAACTTCGGCAAGATGGGCACGA	blaSPM 1	70	MBL fold metallo-hydrolase [Derxia lacustris]	78	MBL fold metallo-hydrolase [Derxia lacustris]	78	1
Permafrost thaw	>1311:3:57:7509:9146/1	CCGGGGATACGGCGTCGCCGATATCGGGACTGGCCAGCCGGTAGACTCGATCACGCTCTTTCAGGCTGCTTCCATCAGCAAGCCGGTGTGTGCCGTGGCGGCGATGCGCCTGGG	AmpC	55.88	serine hydrolase [Gemmatimonadetes bacterium]	77	serine hydrolase [Gemmatimonadetes bacterium]	77	1
	>1311:3:60:11936:1346/1	CTGGAGAACGCAATCGCCGGCAATCACGGTGCGATCCGCTTCGCGCACGAAAGAACAAGAGCCGGGAGTGTGGCCGGGAGTGTGGATGATGCGCAGCGACCCTGCAGCGAATTC	blaGOB 7	60	MBL fold metallo-hydrolase [Pyrinomonas methylaliphatogenes]	89	MBL fold metallo-hydrolase [Pyrinomonas methylaliphatogenes]	89	1
	>1311:3:67:16883:1479/1	ATTTCTCGACCCCTGTCTTGATCTGCGGCACCGTCTCCGGCAGTCCGCTGTTAATAAGGATGTTCCCCTCGGGCGTGGTGATCAAATAGGTGGACAAATCATAGGTACCCACCC	blaLRA 17	66.67	subclass B3 metallo-beta-lactamase [Chryseolinea serpens]	72	subclass B3 metallo-beta-lactamase [Chryseolinea serpens]	72	1
Metagenomics of a fresh	>M02233:113:000000000-	GCTGCATTTTCTTTTGAGTTGGTTACAAACACACTTATAAAAAAATACTTTCCATTGGGTAAAAATATAATTCCAATATCATTAACTGCTGCCGTTAAACCTTCTTTGTTA	blaVEB 7	69.49	class A beta-lactamase,	79	class A beta-lactamase,	79	1

water pond in Sheffield UK	ABLCP:1:2110:11117:8515 2:N:0:2	ACCCCGGACGAACCAGTTTTGTGAGCAACAATTGTCCCCTTTGGTAATTGCCCCTTTAACCTGCTAGATCGGAAGAGCGTCGTGTAGGGAAAGAGTGTAGGATAAGGTGTAGAGATCGGGGG			subclass A2 [Sporocytophaga myxococcoides]		subclass A2 [Sporocytophaga myxococcoides]		
	>M02233:97:000000000-ABCTJ:1:2102:25847:13373 2:N:0:1	AATGAATTAAAAATAACTCAATTAACCGGTGACTTTTATATTTTCACAACCTATAAAATGTTCGGAACTAAAAAACAATCTGCAAATGGAATGTATTTGGTTACAGACAGTGGAGTTGTGCTTTTTGACTCACCTTGGGACACAAGTCCTTTCCAACCATTACTTGATAGTATAAAAGTAAGACACCATAAAAAAGTTAGGTCGGAAGAGCGTCGTGTAGGGAAAGAGTGTAGGATAGGG TGTAGATCTC	blaJOHN 1	55.13	BlaB/IND/MUS family subclass B1 metallo-beta-lactamase [Flavobacterium frigoris]	68	BlaB/IND/MUS family subclass B1 metallo-beta-lactamase [Flavobacterium frigoris]	68	1
Cross-soil	HWI-EAS137R_0379:6:13:4642:14613#CTTGTA/1	GACCAGACCCTTCGCAGCGCCTTTTCCGTTTCTTGCGTCTGGTATTTTCAGGCGCTGGCGACACGCGTTGGGTTGGCCCGCTAGATCGGAAGAGCACACG	LCR 1	70.83	Class D beta-lactamase	79	Class D beta-lactamase	79	1
GED_Great_Praire_Iowa_assembly	iowa-corn-3-pass.301914.311_[cov=3]	ACAAGATAGAACTATGGGAGCCTGCGGGTGAGTAGCTTCCCAGATTGAGCATGGTAGCATTAATGTAACTTTAGCGCCAAATATTATCATCATGTTTTTTTGTTTGTCGAGGCTCAGGAATATATTGTTTTGTTGCCTGCTGTTTAGCTGTTGTGTGTTTTCTAATACCAGTATTGCGCAGGTGCATGAGCCTACCCTAATTGACTCTACCTGGGAAAAACCTTATGCCCCTTTTCGTATTGCGGGTAACCTGTATTATGTGGGCACTTATGAGCTTACTTCTTACCTCATCGCTACACCACAAGGGTGCA	blaLRA 19	67.44	Subclass B3 metallo-beta-lactamase [Sphingobacteriales bacterium 46-32]	73	Subclass B3 metallo-beta-lactamase [Sphingobacteriales bacterium 46-32]	73	1
	iowa-corn-3-pass.2688816.429_[cov=10]	GCAGAGCCAGTCGGTAAGCCGTTTGCGGGACCGCTCCGCCAGAACCTCGCCGAAGAGCATCTTTTCGACGCTCTGGATCATGGCGTTCGGTGATGTCGTATCAAGCGGACCGTCTGGACTCACCGTGTTCAATGCAGGCTCCACGCGATCGAGTCGCGTAACTTTGTCACCTATGTCGCGCATGAAAGCGTTGAAGCCACTGGGGCCGCCAAGACTCGTGAGCAATAGATTTCCTGCCGTGTTGTCACTCACCGTGATCGCCGCCTGGCACAGCTCACCCACCGTCATCCCTTCTCCCGTGGCATGCAGACTTGTTACAGGGGCGTATGAAAGCAGCGACTCGCGTCCGTACACGACTCGCCGATCAAGCGACTCCTTGCCCTCATCTACTCGCCTGAGAACCGCTGCGACGGCCAGCGGCTTGAACGT	blaTEM 166	55.63	Class A beta-lactamase [Collimonas arenae]	58	Class A beta-lactamase [Collimonas arenae]	58	1
	iowa-corn-3-pass.4433051.527_[cov=17]	GCTTAACATCTTTTTGTCATAAAATACTTTAAGTAATTCGGTCGAAGCCAAAGGTGTTGCTGTATTTATCTTTTGAGTGTTAAAATCCTTACGCATTGCTTCTTCATTAACTTTTATTACAAAACCATCTCCGACAATTTTATTGATATAAGTTTGAACATTTATTGGTCCGCCAATTAACCTTAATAAAATATCGCAGCCGATATTGTCGCTTTGTGAAACAGTATAAGTAAGAATTTCAGCTAAAGAAAGTTTAATGTTTCCATTCGGATATTTTTCTCTGATAATACTCCATGTGTTAGGAATTACATCGCTTTTTCTAATAAGTATTTTTTGGTTAATATTCAATTCACCCTTGTCCACTTTGTGGAGAACAAATAATGCAACAGGAAACTTAAATACACTCATAAGCGGATAACTCTTATCACCATTAATGCTTAAGGTGTCACTATCTTCAATACTCAAAATAGATACGCCAACATCAGCTTTTTTTGTTGCAATAATATTTTCTATTTGTTGTCTGAGTG	blaVEB 9	50.58	Beta-lactamase [uncultured bacterium]	57	Beta-lactamase [uncultured bacterium]	57	1
